# Deep learning approach to peripheral leukocyte recognition

**DOI:** 10.1371/journal.pone.0218808

**Published:** 2019-06-25

**Authors:** Qiwei Wang, Shusheng Bi, Minglei Sun, Yuliang Wang, Di Wang, Shaobao Yang

**Affiliations:** 1 School of Mechanical Engineering & Automation, Beihang University, Beijing, China; 2 Department of Technology Research, Beijing iCELL Medical Co. Ltd., Beijing, China; Newcastle University, UNITED KINGDOM

## Abstract

Microscopic examination of peripheral blood plays an important role in the field of diagnosis and control of major diseases. Peripheral leukocyte recognition by manual requires medical technicians to observe blood smears through light microscopy, using their experience and expertise to discriminate and analyze different cells, which is time-consuming, labor-intensive and subjective. The traditional systems based on feature engineering often need to ensure successful segmentation and then manually extract certain quantitative and qualitative features for recognition but still remaining a limitation of poor robustness. The classification pipeline based on convolutional neural network is of automatic feature extraction and free of segmentation but hard to deal with multiple object recognition. In this paper, we take leukocyte recognition as object detection task and apply two remarkable object detection approaches, *Single Shot Multibox Detector* and *An Incremental Improvement Version of You Only Look Once*. To improve recognition performance, some key factors involving these object detection approaches are explored and the detection models are generated using the train set of 14,700 annotated images. Finally, we evaluate these detection models on test sets consisting of 1,120 annotated images and 7,868 labeled single object images corresponding to 11 categories of peripheral leukocytes, respectively. A best mean average precision of 93.10% and mean accuracy of 90.09% are achieved while the inference time is 53 ms per image on a NVIDIA GTX1080Ti GPU.

## Introduction

As a standard practice in clinical medicine, microscopic examination of peripheral blood plays an important role in the field of diagnosis and control of major diseases. It is uniquely capable of discerning clinically relevant morphologic features of hematopoietic cells, including abnormal white blood cells (WBCs, also known as leukocytes) in lymphoma, leukemia, dysplasia and other diseases [1–2]. However, up to now, gold-standard morphologic profiling of blood cells relies heavily on manual smear processing techniques and visual inspection with limitations from quality-control and economic scalability [3]. Blood smear preparation and interpretation are thought to be negatively affected by observer bias, slide distribution errors, statistical sampling error, recording errors, and also involve labor-intensive processes that require highly skilled technicians [4–5]. As such, there has been considerable interest in developing systems for automated classification of digital images of peripheral blood smears with high sensitivity and specificity.

**Fig 1:**
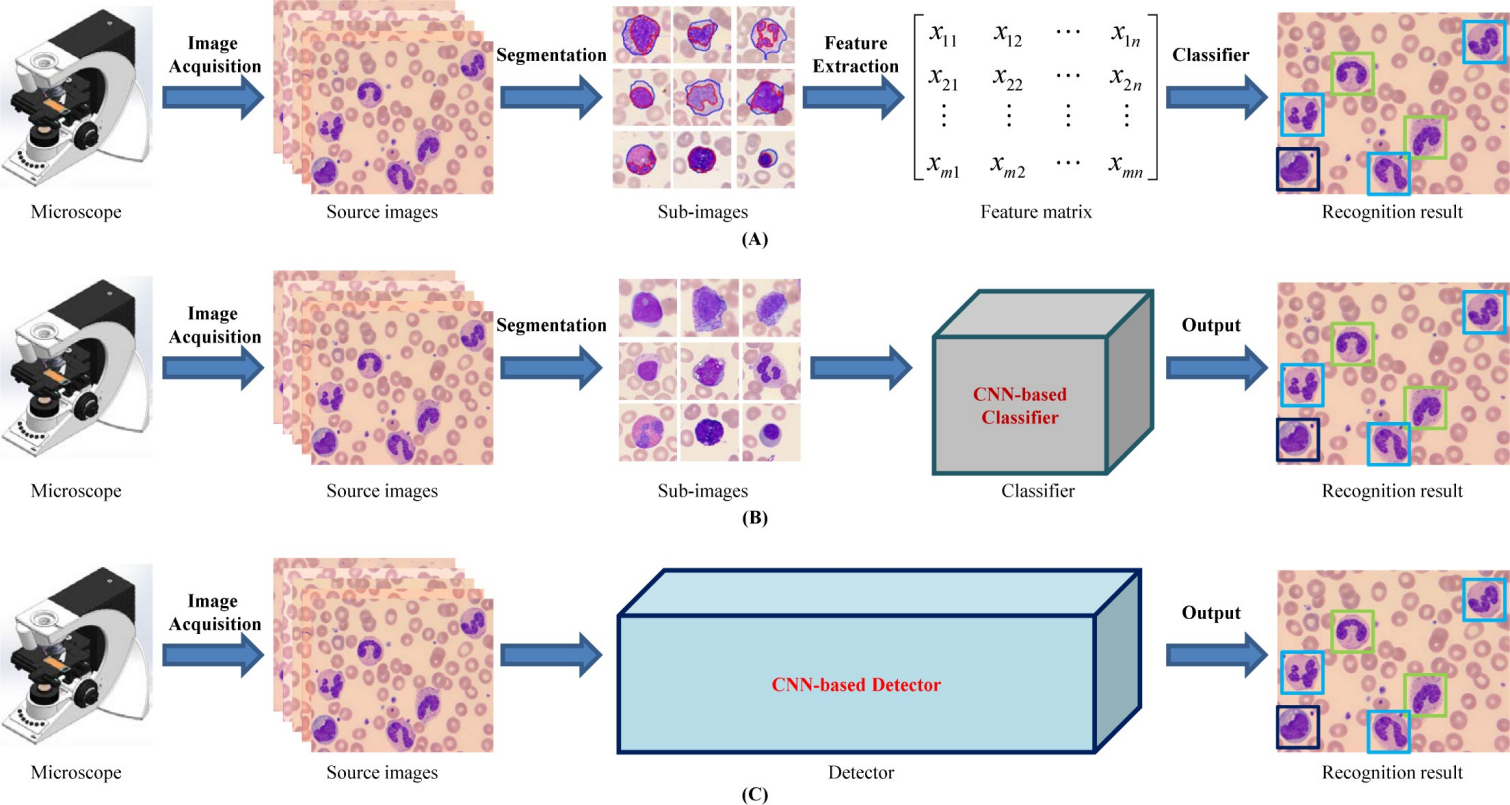
Pipelines of peripheral leukocyte recognition. (A) treat leukocyte recognition as traditional feature engineering: segmentation, feature extraction & selection by manual and then classifier based on the feature matrix; (B) treat leukocyte recognition as object classification: get patches containing leukocyte candidates from original image by manual or segmentation approaches, and then feed these patches into CNN-based deep learning classifier to output the leukocyte types; (C) treat leukocyte recognition as object detection: feed the original images into CNN-based deep learning detector, and then output the leukocyte types and the corresponding locations.

Traditionally, researchers have made efforts to automated morphologic leukocyte differential count. They have used shallow machine learning models that rely on the input derived in a way similar to analysis by morphologists. Such attempts quantify relevant features which are extracted from digital images to serve as the input of prediction algorithms (see Fig 1A). The common shallow machine learning approaches implemented in leukocyte classification generally include *Artificial Neural Networks* (*ANNs*) [6–7], *Supported Vector Machine* (*SVM*) [6, 8–11], *Naive Bayes Classifier* [12, 13], *Linear Discriminate Analysis* (*LDA*) [14, 15] and *Multi-Layer Perceptron* (*MLP*) [7, 16]. To obtain high classification performance, lots of studies focus on image pre-processing [6], object segmentation [17], and feature extraction & selection [18], which are the preconditions of the classification models. Under strictly controlled conditions such as in [16] or by using small datasets such as in [7, 12], the traditional leukocyte recognition systems often achieve high classification accuracy.

Generally speaking, traditional feature engineering systems mainly follow three steps: (I) object(s) segmentation from background; (II) effective or distinctive features extraction by manual; (III) classifier design. These shallow learning models based on small sample learning can often achieve good performance but heavily rely on segmentation accuracy and effectiveness of features. That is to say, as long as one step above goes wrong, the performance of the whole system will be affected. And it must be pointed out that robust and reliable segmentation itself is a non-trivial problem. Any over/under segmentation errors have a negative effect on the overall system performance. The feature extraction by manual is also a very crucial step, and both the quantity and the quality are sensitive to the final performance of system accuracy. To explore leukocyte recognition method with image segmentation free, Dan et al.[19] characterized white blood cells with local descriptors and applied the well-known bag of words as a pooling mechanism. In this research, SIFT (Scale-Invariant Feature Transform [20]) detector, oFAST (Oriented Features from Accelerated Segment Text [21]) detector and CenSurE (the CENter SURround Extrema [22]) detector were employed to obtain key points or interest points descriptors so that these local descriptors can represent for 5 leukocyte types (neutrophil, eosinophil, basophil, monocyte and lymphocyte). But the classification accuracy was unsatisfactory, especially for eosinophil and basophil.

All of the above drawbacks of traditional recognition systems lead to an urgent necessity to develop more adaptive and practical high-performance recognition systems. Then, researches on leukocyte recognition based on deep learning are gradually emerging.

The deep learning technologies have shown impressive performance in various vision tasks such as image classification, object detection and semantic segmentation. The core of the deep learning technology is that the path of the feature extraction is not designed by human engineers but learned from data using a general-purpose learning procedure instead. In the field of deep learning, the convolutional neural networks (CNNs) has achieved excellent performance in image analysis. It's relatively easy to build up an end-to-end model for using CNN-based pipeline. Moreover, CNN-based deep learning architecture make it possible to avoid complicated hand-crafted features design and achieve desired performance. Therefore, the CNN-based approaches have been rapidly emerging in leukocyte recognition. Zhao et al. [23] proposed an automatic detection and classification system for WBCs from peripheral blood images, where WBCs were detected in terms of the location of nucleus of leukocyte, and CNN architecture (5 convolutional layers and 2 pooling layers) were designed to extract features in high level. This pipeline gave a more valuable idea to deal with the problem of leukocyte recognition by combining detection and classification of WBCs together, but WBC types involved in the research were limited to five common types and the accuracy for some types (eosinophil 70% and lymphocyte 74.8%) still need to be improved. Shahin et al. [24] proposed CNN-based deep learning architecture for 5 mature WBCs (basophil, eosinophil, lymphocyte, monocyte and neutrophil) recognition and achieved a higher classification accuracy more than the traditional WBCs identification approaches. Besides, in bone marrow smear images, Choi et al. [25] employed an automated white blood cell differential counting system using a dual-stage convolutional neural network. The dual-stage CNN architecture classified images into 10 types of myeloid and erythroid maturation series, and achieved excellent performance. Moreover, based on deep residual learning theory and medical domain knowledge, Qin et al. [26, 27] presented a fine-grained leukocyte classification method for microscopic images. This proposed deep residual neural network was tested on microscopic image dataset with 40 leukocyte categories, and achieved desired results. From the above studies, we can notice that the research object ranged from 5 types of peripheral blood to 10 or 40 types of bone marrow, and the number of training set ranged from 2174, 2551 to 92480 images. Although deep CNN and the traditional machine learning methods have shown good results in the classification of blood cell images, they are unable to fully exploit the long-term dependence relationship between certain key features of images and image labels. To solve this, an CNN-RNN (Recursive Neural Network) framework [28] was designed to deepen the understanding of image content and learn the structured features of image.

To sum up, most methods mentioned above were designed from the perspective of image classification, i.e., treating WBC recognition as classification task [24–27, 29–30], which must ensure that there exist object candidates in the input image by segmentation, and the number of objects do not exceed one by cropping image manually or complicated segmentation step, as shown in Fig 1B. These classification-task-driven methods are generally aimed at recognize five types of mature leukocytes commonly seen in peripheral blood, and start the classification from the cropped WBCs mages by experts, which results in the inconvenience in real applications.

Generic object detection, aiming at locating object instances from a large number of predefined categories in images, is one of the most fundamental and challenging problems in computer vision [31–32]. Nevertheless, in spite of its potential, to the best of our knowledge, this object-detection-task-driven approach has not been applied on leukocyte recognition problem before. We therefore attempt to deal with the WBCs recognition of multi-object images from the perspective of object detection rather than image classification, hoping to correctly tell what type and where the leukocyte is in the resource image captured directly from the microscope in a fashion manner of end to end, as demonstrated in Fig 1C.

There exist two established series as representatives of deep learning methods: two-stage detection framework, which includes a pre-processing step for region proposal, making the overall pipeline two stage; and one-stage detection framework, or region proposal free framework, which does not separate detection proposals, making the overall pipeline as single stage with the elegant manner of end to end. The typical architectures for the two-stage pipeline include Regions with CNN features (*R-CNN*) [33], Spatial Pyramid Pooling in Deep Convolutional Networks (*SPP-net*) [34], *Fast R-CNN* [35], *Faster R-CNN* [36], Region-based Fully Convolutional Networks (*R-FCN*) [37] and *Mask R-CNN* [38] while *DetectorNet* [39], *MultiBox* [40], *OverFeat* [41], You Only Look Once (*YOLO*) [42], *YOLOv2* [43], *YOLOv3* [44] and Single Shot Multibox Detector (*SSD*) [45] for one-stage pipeline.

Among various above mentioned object detection pipeline, SSD [45] is relatively fast and robust to scale variations because it makes use of multiple convolution layers and combines all predictions from multiple feature maps with different resolutions for object detection. YOLO [42] is a unified detector casting object detection as a regression problem from image pixels to spatially separated bounding boxes and associated class probabilities. As an incremental improvement version of YOLO, YOLOv3 [44] runs significantly faster than other detection methods with comparable performance. Namely, up to now, YOLOv3 has achieved the best trade-off between detection accuracy and computational speed.

Liang et al. [46] treated the urinary particle recognition as object detection and employed Faster RCNN [36] and SSD [45] methods, along with their variants, for urinary particle recognition. And what's more, the result of their study was encouraging. Inspired by this research, we also take the WBCs recognition as object detection task and then try to exploit two well-known CNN-based object detection methods, SSD and YOLOv3, for leukocyte detection. When applying these approaches to WBCs recognition, we adopt the mechanism of deep transfer learning (fine tune corresponding pre-training models rather than from scratch), and conduct extensive experimental analysis to demonstrate the impact of various factors. In detail, when using SSD or YOLOv3, we adjust several parameters, including the scales of default boxes, the size of input images and the backbone net to boost WBCs recognition performance.

## Materials and methods

### Blood sample preparation and digital image acquisition

Our samples were residual peripheral blood obtained from the routine workload of the Clinical Laboratory at Peking University First Hospital and the Peking Union Medical College Hospital. Venous blood was collected into tubes containing K3EDTA as anticoagulant. The samples were analyzed by a cell counter Advia 2120 (Siemens Healthcare Diagnosis, Deerfield, USA) and peripheral blood films were automatically stained with Wright Giemsa in the SP1000i (Sysmex, Japan, Kobe) within 4 hours of blood collection. It should be noted that the materials needed in this study are only blood smears left after the use of above-mentioned hospital routine work. These blood smears do not involve any personal privacy information of patients and their only mission is to provide images as the input of leukocyte recognition algorithm.

The quality of the smears and cell morphology was assessed by hematologists prior to the image processing. The individual cell images for this study had a resolution of 732×574 pixels and they were obtained by the automated blood cell morphology system, *iCELL ME-150* (China Food and Drug Administration-approved for automated image analysis, *iCELL* Medical Company, Beijing, China), where a 100x oil immersion Plan Semi Apochromatic objective (1.30 N.A., 0.2 mm W.D.) was utilized for the acquisition of images with a pixel resolution of 0.13 um. We prepared 500 slides of different subjects randomly from the routine workload of the hospitals above. For each slide, a total of 200 leukocyte candidate images were captured.

### Methods

In this paper, two well-known CNN-based object detection methods, SSD and YOLOv3, were employed for WBCs recognition.

#### Data collection

**Fig 2:**
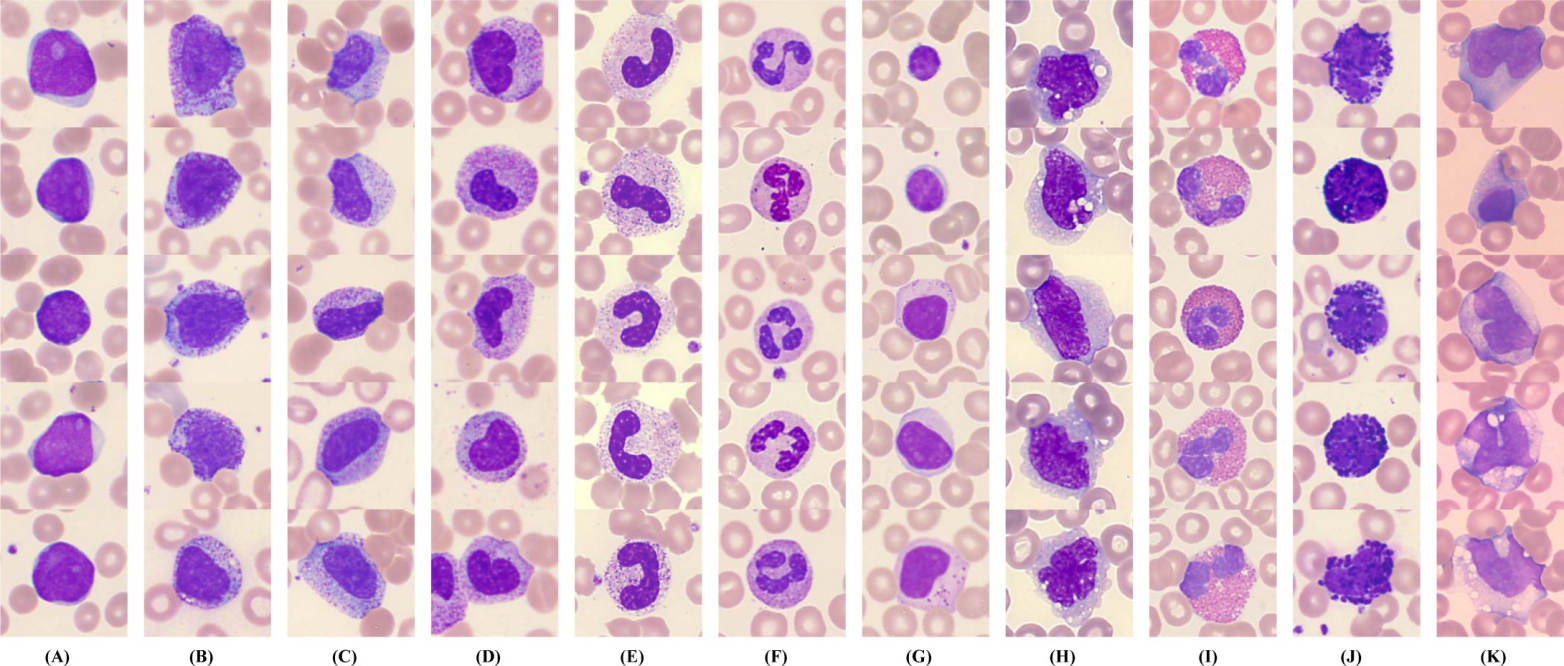
Selected samples of peripheral leukocytes. (A) blast; (B) promyelocyte; (C) myelocyte; (D) metamyelocyte; (E) band neutrophil; (F) segmented neutrophil; (G) lymphocyte; (H) monocyte; (I) eosinophil; (J) basophil; (K) reactive lymphocyte.

In order to perform this study, we firstly established the peripheral leukocyte micro-images database that was labeled with ground truth boxes by more than three clinical experts. All 14,700 annotated color images have a size of 732×574, and include 11 categories of leukocytes, i.e., blast (Blast), promyelocyte (PRO), myelocyte (MYE), metamyelocyte (MET), band neutrophil (bNEU), segmented neutrophil (sNEU), lymphocyte (LYM), monocyte (MO), eosinophil (EO), basophil (BA) and reactive lymphocyte (rLYM). Fig 2 shows 11 categories of peripheral leukocytes from our database, each of which includes various shapes. In fact, we added four non-WBC types, i.e., nucleated red blood cell (NRBC), giant platelet (gPLT), smudge (SM) and artefact (Artefact), for the model unified training. But the recognition and evaluation of the four non-WBC types were not covered in this paper.

**Fig 3:**
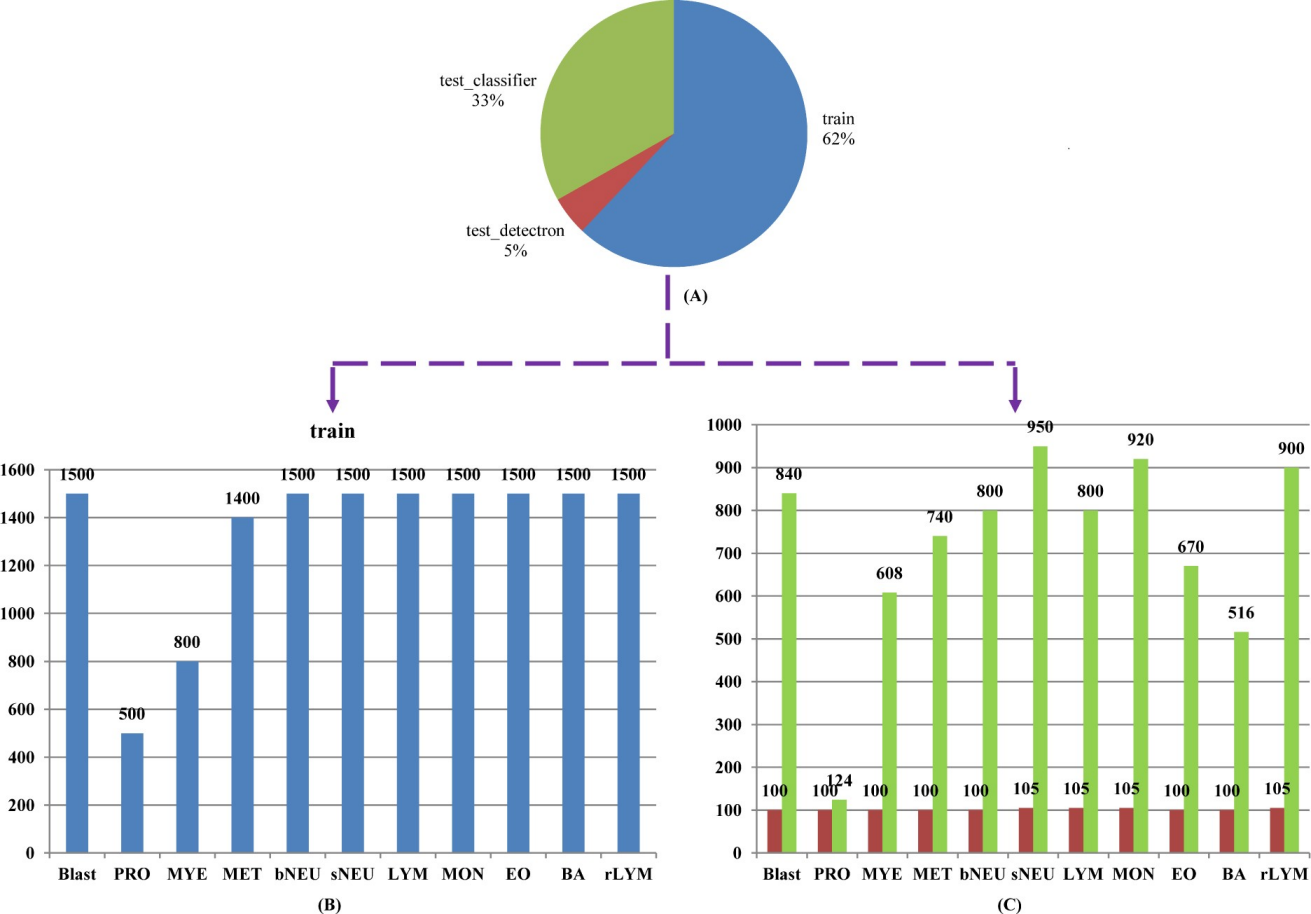
Dataset organization and categories distribution. (A) proportion of train/detection test/classification test sets; (B) train set (14700images, 11 types of peripheral leukocytes) distribution; (C) detection test set (reddish-brown chart, 1120 images) and classification test set (green chart, 7868 images) distribution.

Our 14,700 annotated images have a total of 19,258 ground truth boxes, which contain 3,541 for Blast, 546 for PRO, 1,454 for MYE, 1,032 for MET, 1,466 for bNEU, 2,666 for sNEU, 1,819 for LYM, 1,689 for MO, 1,634 for EO, 1,541 for BA and 1,870 for rLYM. From the 14,700 images, 1,120 images were selected at random to make up 7/100 as the test set, and the remaining as the train set. Besides, in order to better evaluate the classification performance of the models, we collected 7,868 images (without duplicate images with train set) for classification test set, where every image contains single object. Fig 3 demonstrates the details of dataset organization and categories distribution. The top pie chart (Fig 3A) shows how selected images are organized into train/detection test/classification test sets. The bottom bar graphs (Figs 3B and 6C) display detailed leukocyte distribution for the imbalanced database.

#### Leukocyte recognition based on SSD

As a single-shot multibox detector for multiple categories, SSD method can be decomposed into a truncated base network and several auxiliary convolutional layers used as feature maps and prediction. SSD has achieved excellent performance by the trade-off between detection accuracy and speed. Different from Faster R-CNN, SSD increases detection speed by removing the region proposal generation and the feature re-sampling stages. Unlike YOLO, SSD improves detection quality by applying a set of small convolutional filters (kennel size: 3×3) to multiple feature maps to predict confidences and boxes locations for multi-scale categories.

**Fig 4:**
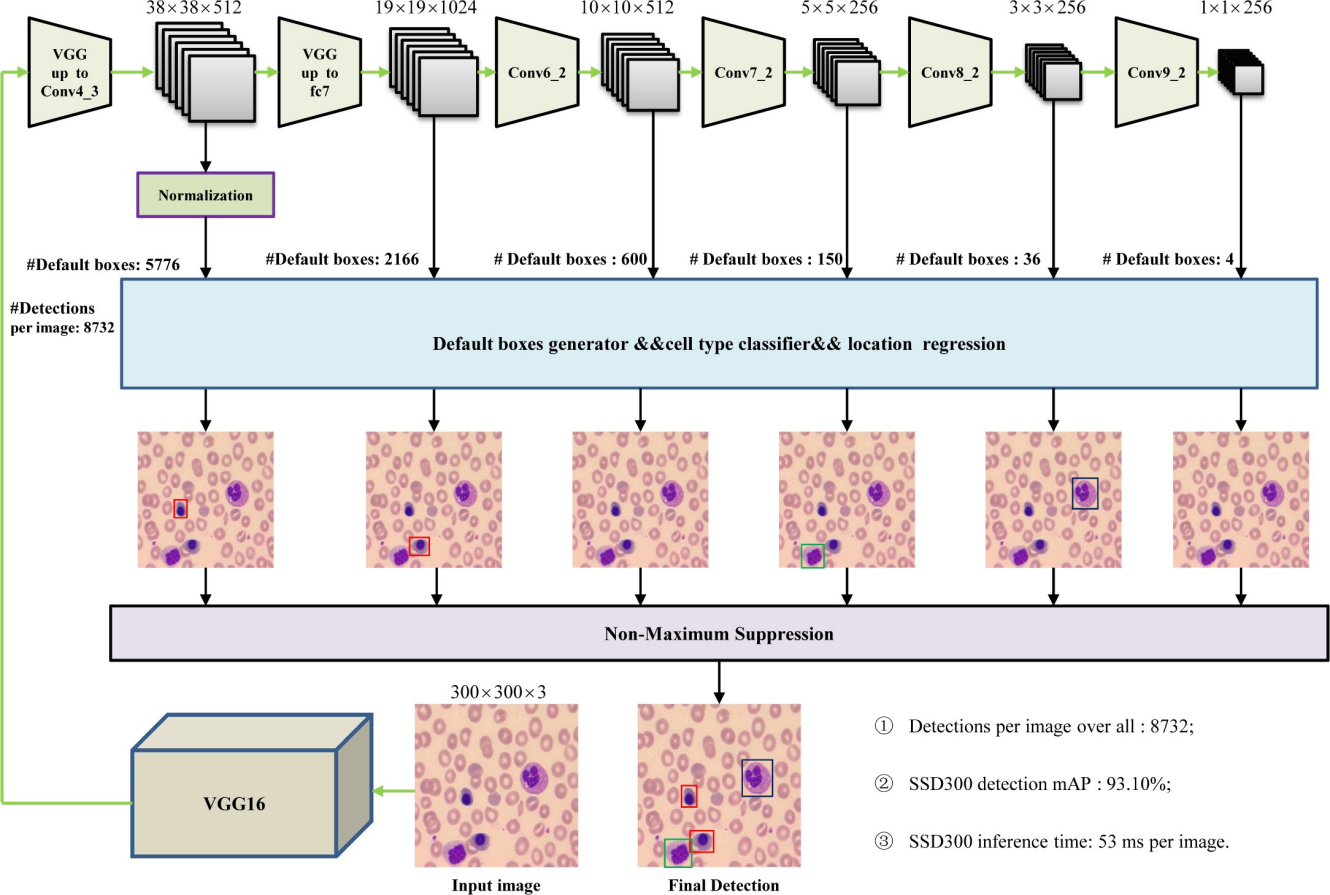
SSD architecture. To multi-scale feature maps for detection, several feature layers (Conv6_2, Conv7_2, Conv8_2 and Conv9_2) were added to the end of base network (VGG16), where the larger size feature maps, such as Conv4_3, were used to detect small size leukocytes while the smaller size feature map to detect the large size.

Fig 4 shows the detailed pipeline of SSD300 architecture. Conv4_3, fc7(convolution layer), Conv6_2, Conv7_2, Conv8_2 and Conv9_2 were used to generate default boxes, and predict both location and confidence for every default box. 8732 detections per image were resulted in and the final detection was output by non-maximum suppression strategy. The more detailed information of SSD can be referred in the literature [45].

When training SSD300×300, we fine-tuned a pre-trained model with Stochastic Gradient Descent (SGD) for 1000 mini-batch iterations, with a mini batch size of 10 on 1 GPU, a momentum of 0.9 and a weight decay of 0.0005. By default, we adopt the multistep learning rate policy with a base learning rate of 0.001, a step value of [14,700, 147,000, 294,000, 735,000] and a gamma of 0.1.

As we know from the literature [45], SSD discretizes the output space of bounding boxes into a set of default boxes over different aspect ratios and scales per feature map location. In order to relate these default boxes from different feature maps to corresponding receptive fields, the authors in the literature [45] designed a scale strategy that regularly but roughly responses specific boxes to specific areas of the image, where the lowest layer has a scale of *S*_*min*_ and the highest layer has a scale of *S*_*max*_, and all layers in between are regularly spaced.

Considering that there may exist some small leukocyte candidates in cell images, we adjusted empirically the scales parameter of default boxes when training SSD300×300. From experimental results in Table 1, we can see that the reduced minimum scale from 0.2 to 0.1 (the maximum scale of 0.9 remained unchanged) decreases mAP by 10%.

**Table 1:** Comparison of detection results using SSD and YOLOv3 series models.

Model	Smin	Smax	mAP	Blast	PRO	MYE	MET	bNEU	sNEU	LYM	MON	EO	BA	rLYM
**SSD300×300**	0.2	0.9	**0.931**	**0.970**	**0.870**	0.862	**0.881**	**0.982**	0.970	0.884	**0.914**	**1.0**	**0.993**	0.913
	0.1	0.9	0.831	0.882	0.654	0.745	0.663	0.853	0.928	0.849	0.806	0.985	0.973	0.800
**SSD512×512**	0.2	0.9	0.687	0.651	0.453	0.682	0.499	0.777	0.741	0.409	0.756	0.907	0.891	0.790
**YOLOv3_320×320 -**			0.925	0.961	0.826	0.860	0.841	0.955	0.968	0.937	0.912	0.999	**0.993**	**0.920**
**YOLOv3_416×416**		-	0.921	0.958	0.824	0.862	0.826	0.947	0.968	**0.947**	0.894	0.999	0.987	0.915
**YOLOv3_608×608**		-	0.919	0.962	0.814	**0.864**	0.829	0.961	**0.974**	0.930	0.904	0.999	0.987	0.881
**YOLOv3-tiny_416×416 -**			0.772	0.941	0.469	0.701	0.372	0.758	0.915	0.871	0.674	0.993	0.982	0.815

SSD300×300 has an input size of 300×300, SSD512×512 increases it to 512×512, and YOLOv3 is the 3rd version of YOLO architecture, with the different backbone net (YOLOv3-tiny) and different input size: 320×320, 416×416, and 608×608.

Generally, increasing the size of input images can improve detection precision, especially to small objects. We tried to increase the input size from 300×300 to 512×512. Here we trained SSD512×512 only once, with a minimum scale of 0.2 and a maximum scale of 0.9. Unfortunately, we just obtained a poor 68.7% mAP (Table 1), because of decreasing the batch size setting from 10 to 4 to run this model owing to limited GPU resources (*GPU NVIDIA GTX 1080Ti*, 11 GB). We tend to argue that appropriate hyper parametric configuration would helps to achieve better results if sufficient GPU resource.

#### Leukocyte recognition based on YOLOv3

YOLOv3, another end-to-end and one-stage detector, is much better than SSD variants and comparable to state-of-the-art models on the metric of average precision with the intersection over union (IoU) of 0.5. YOLOv3 continues the main patter of the former YOLO and YOLO9000 dealing with object detection problem by a regression pipeline. But different from previous YOLO versions, the backbone of YOLOv3 is Darknet 53 [47], which includes 53 layers of convolution, and Resnet short cut connections to avoid the disappearance of gradients. In the prediction stage, the FPN (Feature Pyramid Network) uses three scale feature maps, where small feature maps provide semantic information, and large feature maps provide finer-grained information. Small feature maps are fused by upper sampling and large scale. Besides, independent logistic classifiers rather than softmax is employed in YOLOv3 architecture and binary cross-entropy loss for the class predictions is used in training stage.

**Fig 5:**
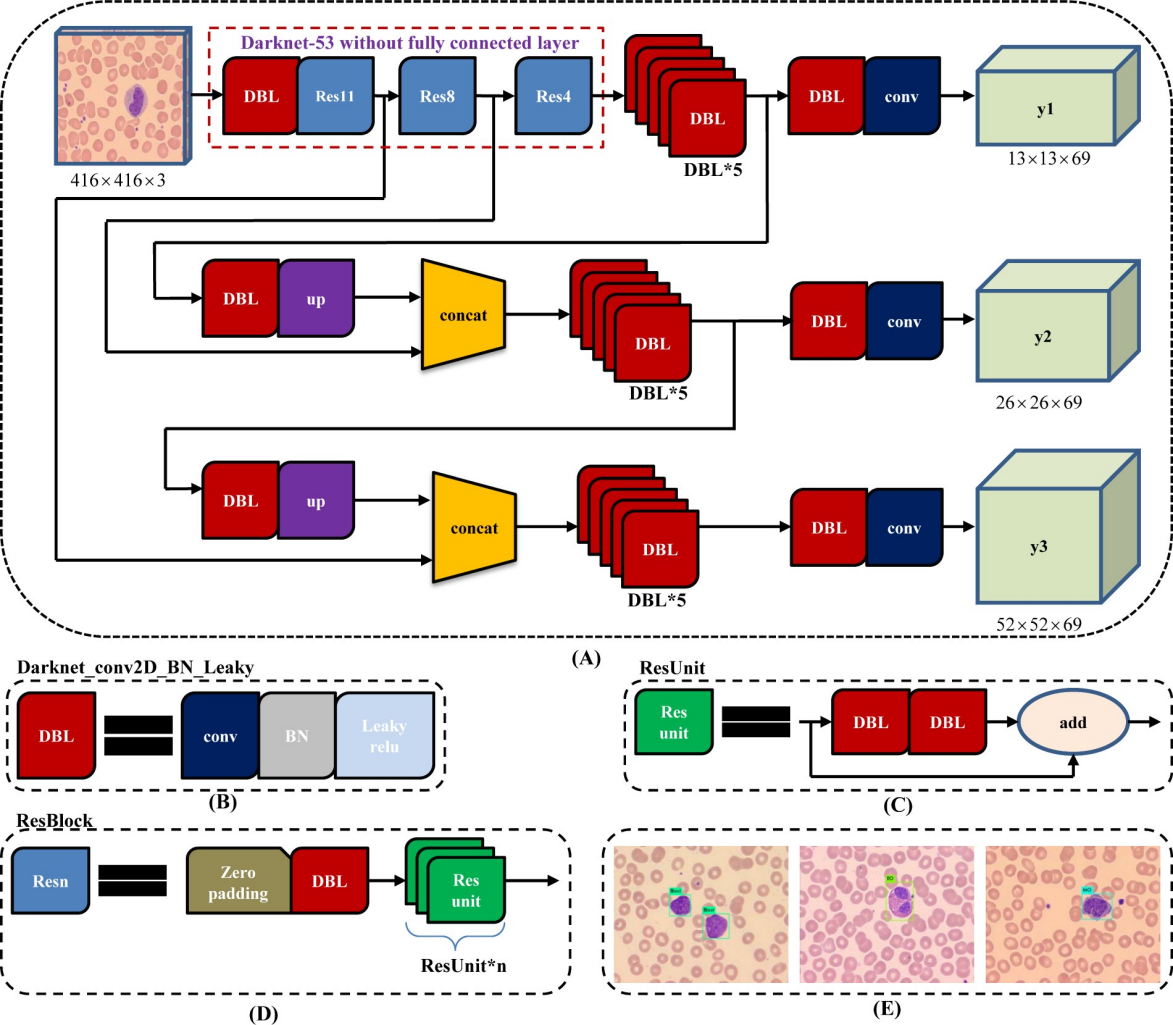
YOLOv3 architecture. (A) YOLOv3 pipeline with input image size 416×416 and 3 types of feature map (13×13×69, 26×26×69 and 52×52×69) as output; (B) the basic element of YOLOv3, Darknet_conv2D_BN_Leaky ("DBL" for short), is composed of one convolution layer, one batch normalization layer and one leaky relu layer.; (C) two "DBL" structures following with one "add" layer leads to residual-like unit ("ResUnit" for short); (D) several "ResUnit" with one zero padding layer and "DBL" structure forward generates residual-like block, "ResBlock" for short, which is the module element of Darknet-53; (E) some detection results of peripheral leukocyte using YOLOv3 approach, resize the 732×574 images to 416×416 size as input.

The size of input images should be an integer multiple of 32 (such as 320×320, 416×416 and 608×608), owing to the total of 5 steps for down sampling operation leading to the most stride is 32. As demonstrated in Fig 5, YOLOv3 employs darknet-53 without the last fully connected layer as backbone net, and use Resnet short cut connections. Besides, for every image, three types of detection are conducted with corresponding different receptive fields, where the 32-fold down sampling is suitable for large object detection (Fig 5A, y1), the 16-fold for middle size (Fig 5A, y2), and the 8-fold for small size (Fig 5A, y3).

**Fig 6:**
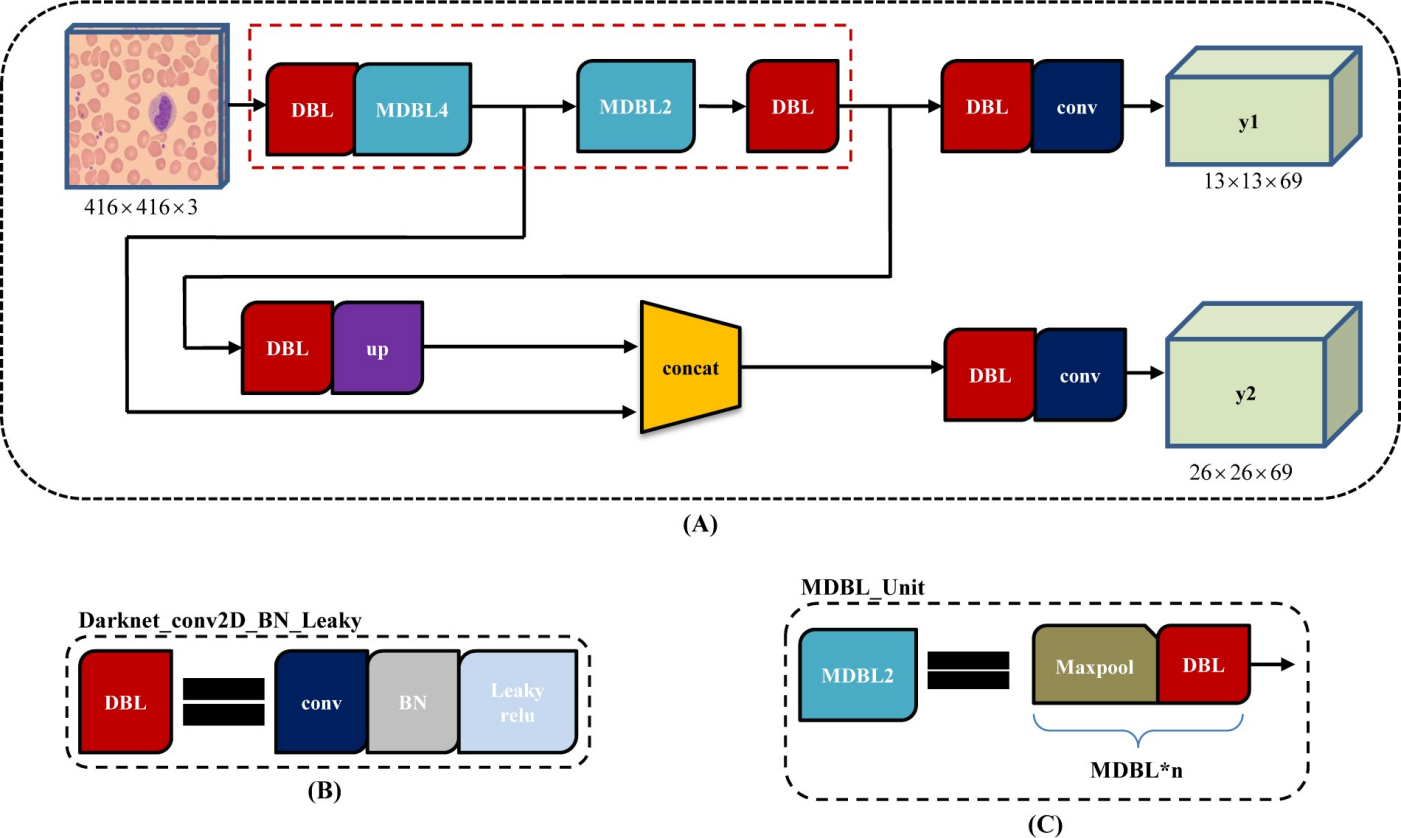
YOLOv3-tiny architecture. (A) the pipeline of YOLOv3 with 2 branch outputs, y1 and y2; (B) the basic element, composed of one convolution layer, one batch normalization layer and one leaky relu layer; (C) one maxpool layer and one "DBL" structure form into "MDBL_Unit" for short.

YOLOv3-tiny, a simplified version of YOLOv3, is widely used because it runs faster and takes less memory. As showed in Fig 6A, compared with YOLOv3, Yolov3-tiny finally has two branch outputs for prediction. Corresponding to 416x416 input image, the size of feature maps is 13×13 and 26×26, respectively. In addition, Yolov3-tiny adopt "MDBL_Unit" (Fig 6C) in its backbone net rather than "ResBlock" (Fig 5D).

For YOLOv3 training we fine-tune a pre-trained model, darknet53.conv.74, with a mini batch size of 64, max_batches of 50,200, and subdivisions of 16 on 1 GPU, a momentum of 0.9 and a weight decay of 0.0005. By default, we adopt the multistep learning rate policy with a base learning rate of 0.0001, a step value of [10,000, 35,000] and the learning rate scales of [0.1, 0.1].

Choosing the appropriate input size according to the target size often makes the detection algorithm more effective. Here, we selected the input sizes as following: 320×320, 416×416 and 608×608.

Compared with YOLOv3, Yolov3-tiny finally has only two branch outputs for prediction, and directly uses "MDBL_Unit" structure rather than "ResBlock", which leads its architecture more shorter and thinner. To achieve a better tradeoff between performance and cost, we tried to employed YOLOv3-tiny for comparison.

#### Evaluation metrics

The trained network was tested on the test dataset and generally could be assessed quantitatively through the metrics: precision, recall and accuracy as follows,

$$Precision=\frac{T_{P}}{T_{P}+F_{P}}$$ (1)

$$Recall=\frac{T_{P}}{T_{P}+F_{N}}$$ (2)

$$Accuracy=\frac{T_{P}+T_{N}}{T_{P}+F_{P}+T_{N}+F_{N}}$$ (3)

where *T_P_* is the number of true positive classifications, *T_N_* the number of true negatives, *F_P_* is the number false positive classifications, and *F_N_* the number of false negatives.

By default, PASCAL-style Average Precision (AP) at a single IoU threshold of 0.5 and mean Average Precision (mAP) were used to evaluate our detection results. And PR (precision versus recall) curves for every leukocyte type were created to analyze detection performance. As to classification test set, the confusion matrix for class-wise performance was generated and then the mean accuracy was computed.

## Results and discussion

The proposed methods based on SSD and YOLOv3 were implemented using the Caffe [48] and Darknet [47] framework respectively. And the *CUDA* 8.0 toolkit with the *cuDNN* 6.0 library on 64bits Ubuntu16.04 operation system was configured. All experiments were performed with a configuration of *CPU i7-6700* (3.40 GHz), *RAM 16 GB*, *GPU NVIDIA GTX 1080Ti* (11 GB) and Python 2.7.

As mentioned, in training stage, we adopted the transfer learning mechanism: first initialized CNN frameworks with pre-trained models based on ImageNet [49] dataset, then fine-tuned all layers of the network using our own datasets.

### Training loss and test performance

**Fig 7:**
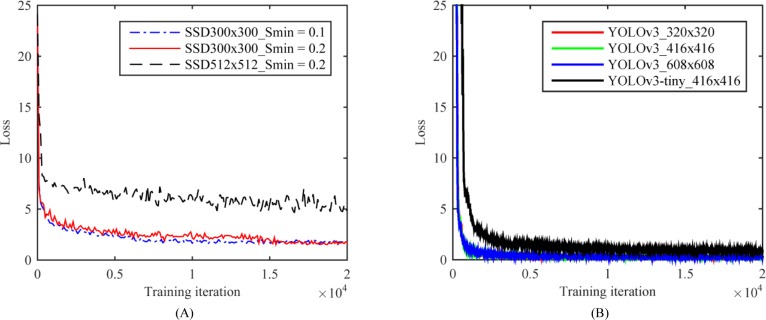
Convergence studies on train loss with respect to the number of iterations. (A) Effect of different scales of default boxes by changing hyper parameter *Smin* and input size on SSD model convergence; (B) Effect of different input size and backbone net on YOLOv3 model convergence.

In order to test the performance of the CNN-based deep learning network model, we conducted systematic convergence studies with respect to the number of iterations. Here we show some representative results. For the same train and test set, we evaluated the training loss of SSD series and YOLOv3 series respectively, as displayed in Fig 7. We find that the training loss of both SSD model and YOLOv3 model decay with the increasing number of training iterations. Moreover, another significant observation in Fig 7 is that the training error of SSD300×300 model has smaller fluctuation and convergence to smaller value among SSD series (see Fig 7A) while the training error of YOLOv3 achieves smaller convergence value among YOLOv3 series (see Fig 7B). Besides, Table 1 gives the mAP and AP for every leukocyte type between the detection models as described above.

**Fig 8:**
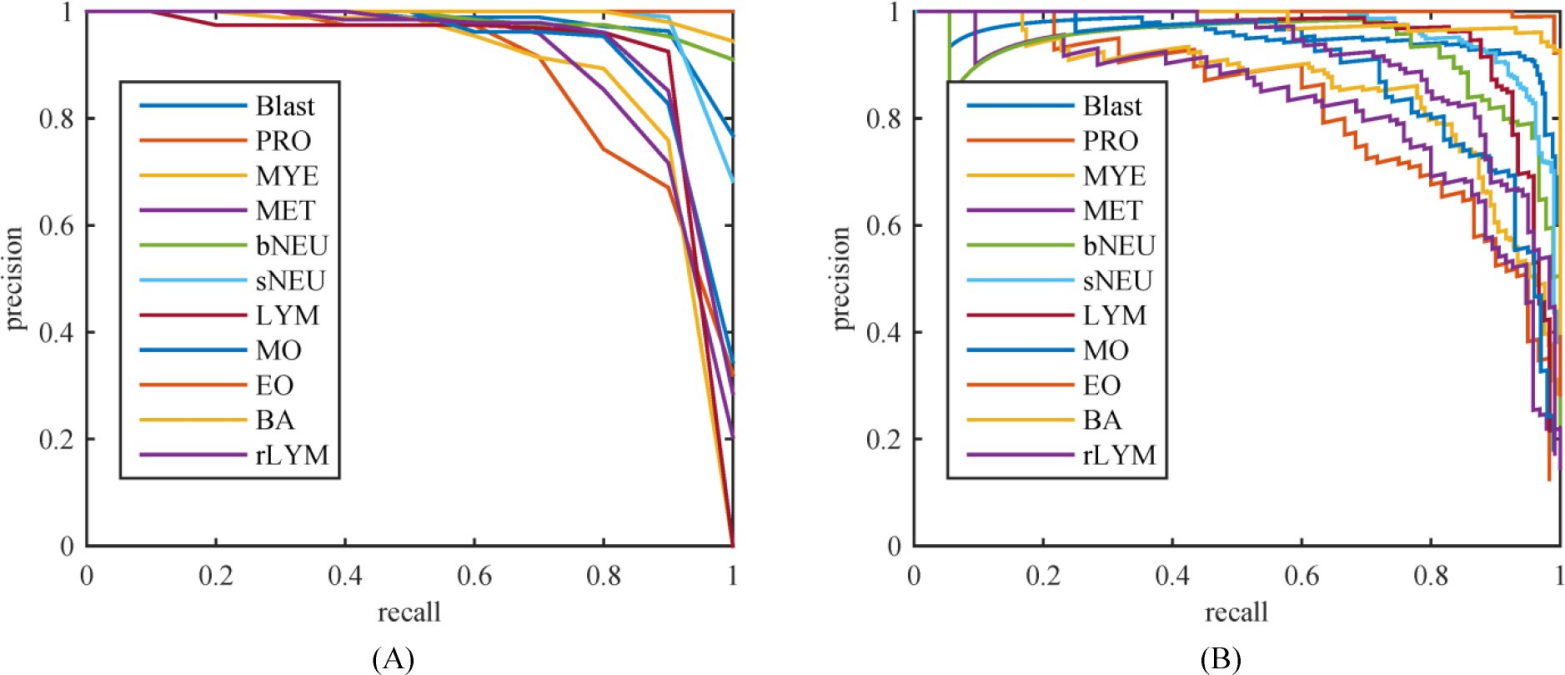
Precision versus recall. (A) SSD300×300_Smin = 0.2 with a mAP of 0.931; (B) YOLOv3_320×320 with a mAP of 0.925.

Table 1 listed the evaluation metrics of the mAP and AP for the SSD series and YOLOv3 series models. Among these models, we chosen top-2 models in mAP, i.e. SSD300×300_Smin = 0.2 and YOLOv3_416×416, and then plotted precision-recall (PR) curves of 11 WBC types respectively, as showed in Fig 8. From Table 1 and Fig 8, one can clearly see that the model of SSD300×300_Smin = 0.2 leads all of the detection models with the mAP of 93.1%, and it outperforms other models for AP of 7 of 11 WBC types, but AP of lymphocyte was lower than that of YOLOv3_414×416 by 6.3%. By the comparison for the precision performance of 11 WBC types, we can get that the mature WBCs in peripheral blood are detected more precisely than the immature ones, as presented in Fig 8. For example, by the most precise model, i.e. SSD300×300_Smin = 0.2, the AP of both eosinophil and basophil are close to 100% while that of promyelocyte, myelocyte, and metamyelocyte maintain at about 87%. However, the abnormal WBCs in peripheral blood, such as blast and reactive lymphocyte, achieved desired precision score. This indicates that our best model has a certain ability to detect some abnormal cells.

For further analysis between SSD series models, SSD512×512 obtained the lowest precision performance because of the limited GPU computing resource and inadequate hyper parameter optimization. At the same time, we should also note that widening the scale distribution range of default boxes by changing the parameter *Smin* from 0.2 to 0.1 is invalid for the train and test sets because in terms of the mAP and AP for every leukocyte type, the model of SSD300×300_Smin = 0.2 is better than that of SSD300×300_Smin = 0.1. We argue that the reason for this result lies in that these 11 types of leukocytes collected for training have a relatively narrow size distribution.

As to YOLOv3 series models in Table 1, given the same input image size, the precision performance of YOLOv3-tiny was far below that of YOLOv3 model (mAP of 0.772 versus that of 0.921). We argue that the reason lies in the YOLOv3-tiny's backbone net, where more shorter and simplifier architecture rather than residual style block and 3-layer multi-scale feature map output was designed. However, from another aspect, with the same configuration and same input size, the time consumption of YOLOv3-tiny was only 16% of that of YOLOv3, and the inference time nearly 50% as well, as listed in Table 2. In addition, the input image size also influences the model performance more or less. The precisions of YOLOv3 series models were comparable, and that of YOLOv3_320×320 was slightly better. This also reflects to a certain extent that YOLOv3 method is not so sensitive to the size of input image for our leukocytes datasets.

**Table 2:** Comparison of time consuming using different models.

**Models**	**mAP**	**Training time (h)**	**Inference time (ms)**
SSD300×300_Smin = 0.1	0.831	101.3	53
SSD300×300_Smin = 0.2	0.931	101.1	53
SSD512×512	0.687	123.2	100
YOLOv3_320×320	0.925	60	14
YOLOv3_416×416	0.921	60.2	23
YOLOv3_608×608	0.919	61	43
YOLOv3_tiny_416×416	0.772	9.5	14

The second column is the performance value of mAP. The third column is the time (in hours) required to train the model with a CPU i7-6700 (3.40 GHz), RAM 16 GB, GPU NVIDIA GTX 1080Ti (11 GB) while the last column is the average time (in ms) used by the model for each image inference with GPU NVIDIA GTX 1080Ti.

### Inference time

In general, a good detector is often regarded as a compromise between precision and detection speed. Here the mAP, training time and inference time are summarized in Table 2. SSD300×300_Smin reached the first mAP score but the inference time was only 53 ms per image, i.e., 19 frames per second while YOLOv3_320×320 achieved the second mAP (0.6% behind the first one) with a inference time of 14 ms per image (70 frames per second).

### Detection of small leukocytes and dense scenes using selected top-2 models

**Fig 9:**
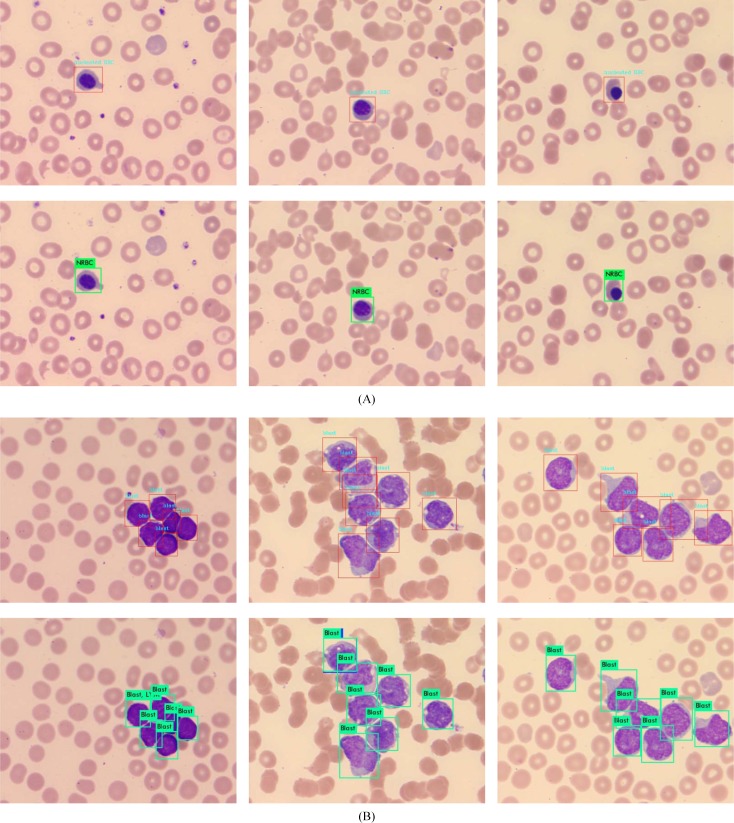
Detection of small cells and dense scenes. (A) NRBC detection where the top was the results of SSD300×300_Smin = 0.2 model and the bottom for YOLOv3_320×320; (B) leukocytes dense scene detection where the top was the results of SSD300×300_Smin = 0.2 model and the bottom for YOLOv3_320×320.

Detection of small objects or dense scenes is always challenging in object detection. Among peripheral nucleated cells, the nucleated red blood cells (NRBC) are usually the smallest ones. The approximate pixel resolution of NRBC is 65×65 out of 732×574 of entire image, with corresponding physical size 8.5×8.5 *um*^*2*^ out of 95×75 *um*^*2*^. Here, we selected 500 images containing NRBC to detect with SSD300×300_Smin = 0.2 and YOLOv3_320×320 models, respectively. None object was missed and all of the 200 images were recognized correctly. Fig 9A shows some NRBC detection results. Besides, we collected a number of acute leukemia blood samples and captured 200 images of dense distribution of WBCs to detect with SSD300×300_Smin = 0.2 and YOLOv3_320×320 models as well. Fig 9B displays the models detection performance for WBCs dense scenes. From the dense leukocyte detection results, both of these top-2 models are in the line with expectation, although some incomplete leukocytes near image boundary were not detected.

### Classification of unseen data set using selected top-2 models

**Fig 10:**
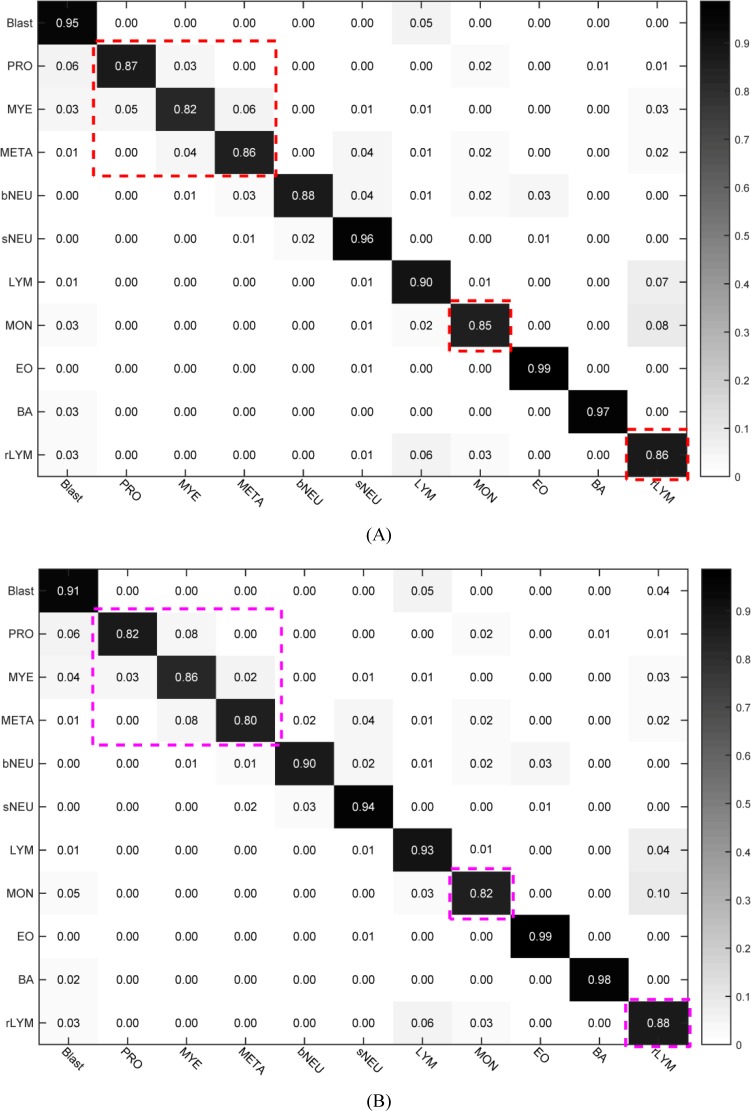
Confusion matrix for selected top-2 models with the 7868 image as classification test set. (A) confusion matrix for SSD300×300_Smin = 0.2 model with the mean accuracy of 90.09%; (B) confusion matrix for YOLOv3_320×320 model with the mean accuracy of 89.36%.

In order to better test the capability of the model, we utilized the test set (Fig 6C) where all of 7868 images covered single object and there existed none duplicate images with train set to test the classification performance of selected top-2 models. The confusion matrixes of SSD300×300_Smin = 0.2 and YOLOv3_320×320 are provided in Fig 10. The mean accuracy of SSD300×300_Smin = 0.2 was 90.09% (see Fig 10A) and that of YOLOv3_320×320 89.36% (see Fig 10B). From Fig 10, we found that it was a bit difficulty to recognize promyelocyte, myelocyte and metamyelocyte. Meanwhile, the accuracy of monocyte or reactive lymphocyte was relatively lower than other types because that quite a part of monocytes were misclassified into reactive lymphocyte type while some reactive lymphocytes were recognized as monocytes and some as blast type. As we know, there exist 3 sub-types of reactive lymphocyte: plasmocyte prototype (I), monocyte prototype (II) and prolymphocyte prototype (III), where the shape of sub-type II and sub-type III are close to monocyte and blast, respectively. That is to say, the ability of fine-grained leukocyte classification needs to be strengthened in the future work.

The above experimental results clearly make out that SSD300×300_Smin = 0.2 slightly outperforms YOLOv3_320×320 in precision (Table 1 and Fig 10), but the latter is more faster. The WBC types of blast, promyelocyte, myelocyte, metamyelocyte and reactive lymphocyte are often regarded as abnormal leukocytes in peripheral blood. And the recognition of these abnormal leukocytes is very significant and valuable for disease diagnosis. In view of the importance of generalization accuracy (90.09% versus 89.36%, Fig 10) in medical applications and the ability to detect abnormal leukocyte of peripheral blood (89.92% versus 88.16%, Table 1), we think SSD300×300_Smin = 0.2 will be a better choice for automated blood cell morphology system.

## Conclusions

To solve the problem of white blood cells differential based on microscopic images, we take peripheral leukocyte recognition as object detection task and select two well-known CNN-based approaches, SSD and YOLOv3, as detection framework. Without significant modification, both SSD and YOLOv3 provide impressive performance. For the sake of detection performance, we carry out extensive experimental analysis to prove the influence of various factors, including backbone net, default boxes scales, and input image size. Experimental results demonstrate that SSD300×300 model achieve a best mAP of 93.10% and generalization accuracy of 90.09% for 11 types of leukocyte with a inference time of 53 ms per image.

In the future work, we will make efforts to expand cell types and fine-grain recognition, especially abnormal leukocyte types, and optimize practical performance in a clinical environment.

## Supporting information

S1 Fig Selected leukocyte recognition examples using SSD300×300_Smin = 0.2 and YOLOv3_320×320 detection models. We show detection results below with the highest confidence scores and intersection over union (IoU) value higher than 0.5. (A)-(M) show the detection results of blast, promyelocyte, myelocyte, metamyelocyte, band neutrophil, segmented neutrophil, lymphocyte, monocyte, reactive lymphocyte, small cells (NRBC) and dense scenes using SSD300×300_Smin = 0.2 model respectively while (N)-(Z) show that using YOLOv3_320×320 model.
(PDF)
