# Supplementary material for: Deep learning approach to peripheral leukocyte recognition
Source: PLoS One. 2019 Jun 25;14(6):e0218808. doi: 10.1371/journal.pone.0218808 (PMC6592546; doi:10.1371/journal.pone.0218808)
Supplement: S1 Fig — We show detection results below with the highest confidence scores and intersection over union (IoU) value higher than 0.5. (A)-(M) show the detection results of blast, promyelocyte, myelocyte, metamyelocyte, band neutrophil, segmented neutrophil, lymphocyte, monocyte, reactive lymphocyte, small cells (NRBC) and dense scenes using SSD300×300_Smin = 0.2 model respectively while (N)-(Z) show that using YOLOv3_320×320 model. (PDF) [file pone.0218808.s001.pdf]

S1 Fig. Selected leukocyte recognition examples using SSD300×300_Smin=0.2 and YOLOv3_320×320 detection models. We show detection results below with the highest confidence scores and intersection over union (IoU) value higher than 0.5. (A)-(M) show the detection results of blast, promyelocyte, myelocyte, metamyelocyte, band neutrophil, segmented neutrophil, lymphocyte, monocyte, reactive lymphocyte, small cells (NRBC) and dense scenes using SSD300×300_Smin=0.2 model respectively while (N)-(Z) show that using YOLOv3_320×320 model.

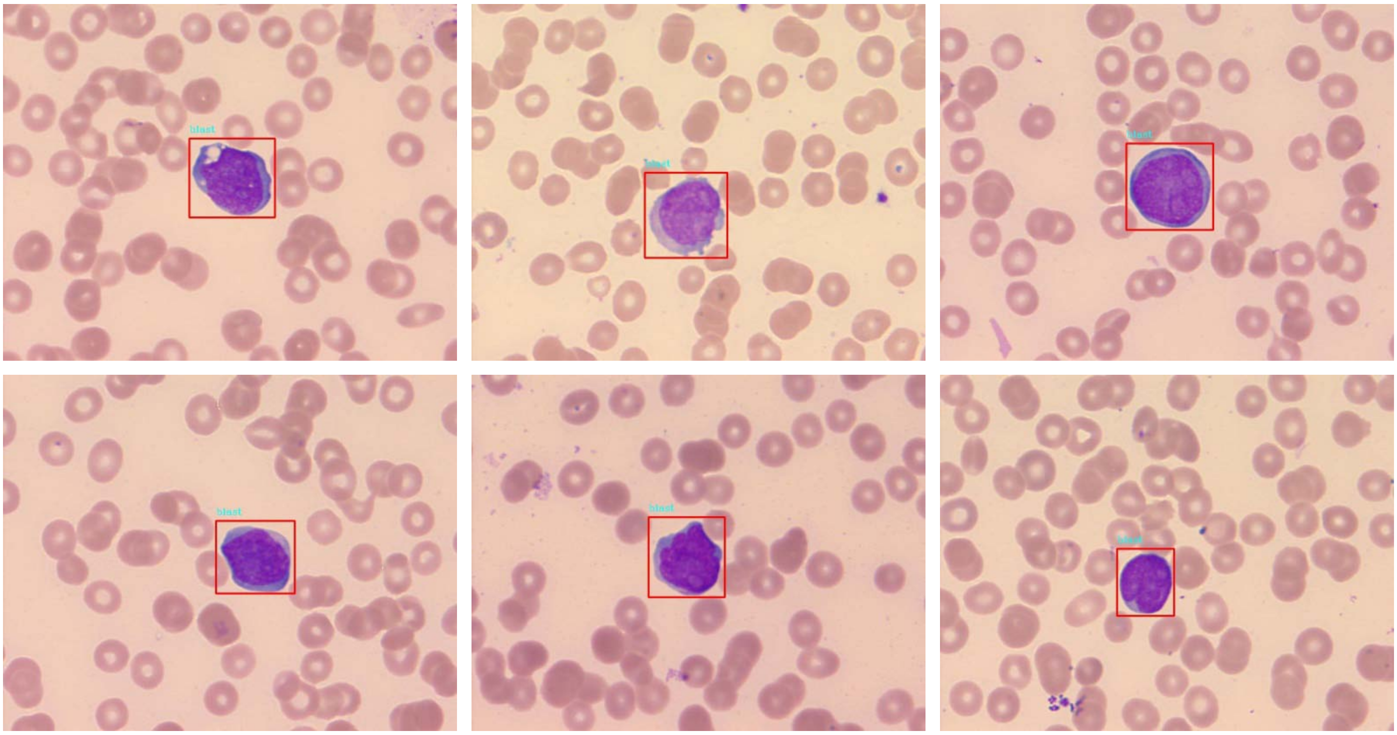

(A) detection results of blast using SSD300×300_Smin=0.2 model

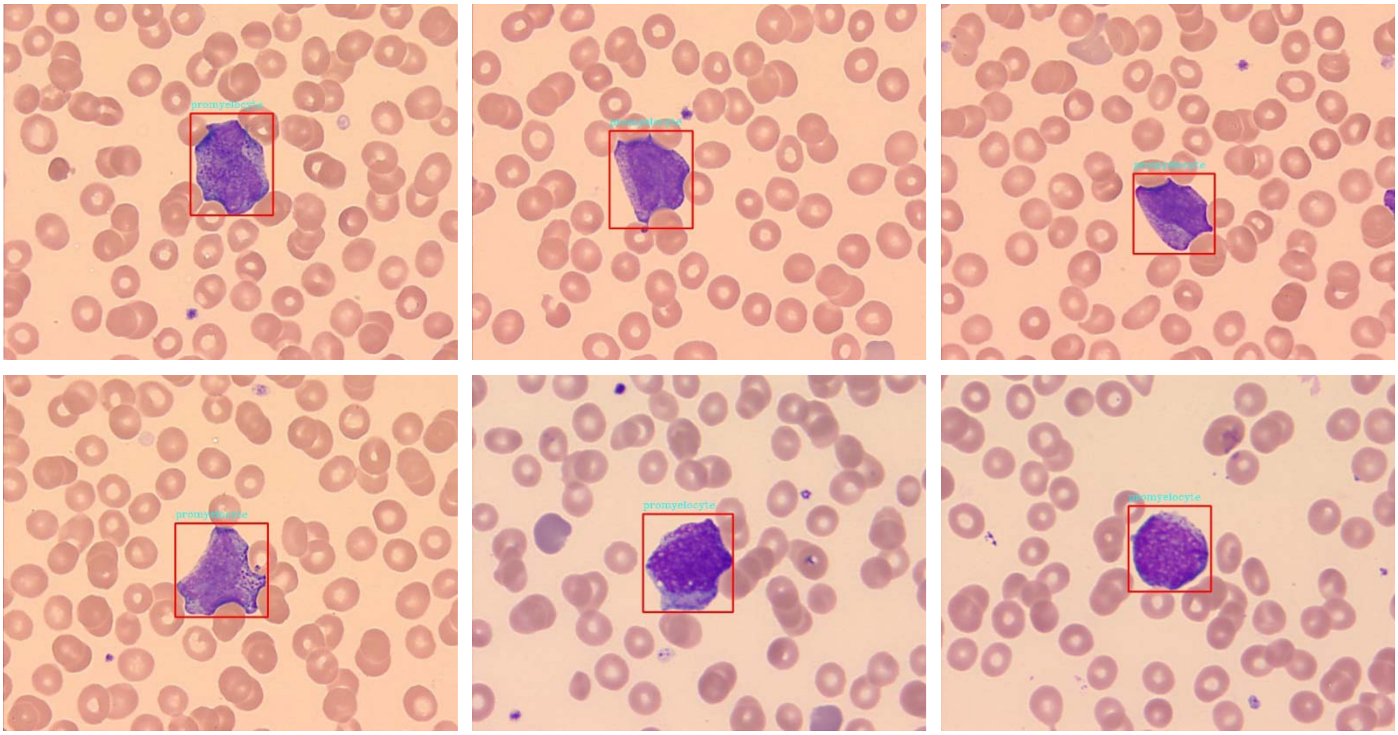

(B) detection results of promyelocyte using SSD300×300_Smin=0.2 model

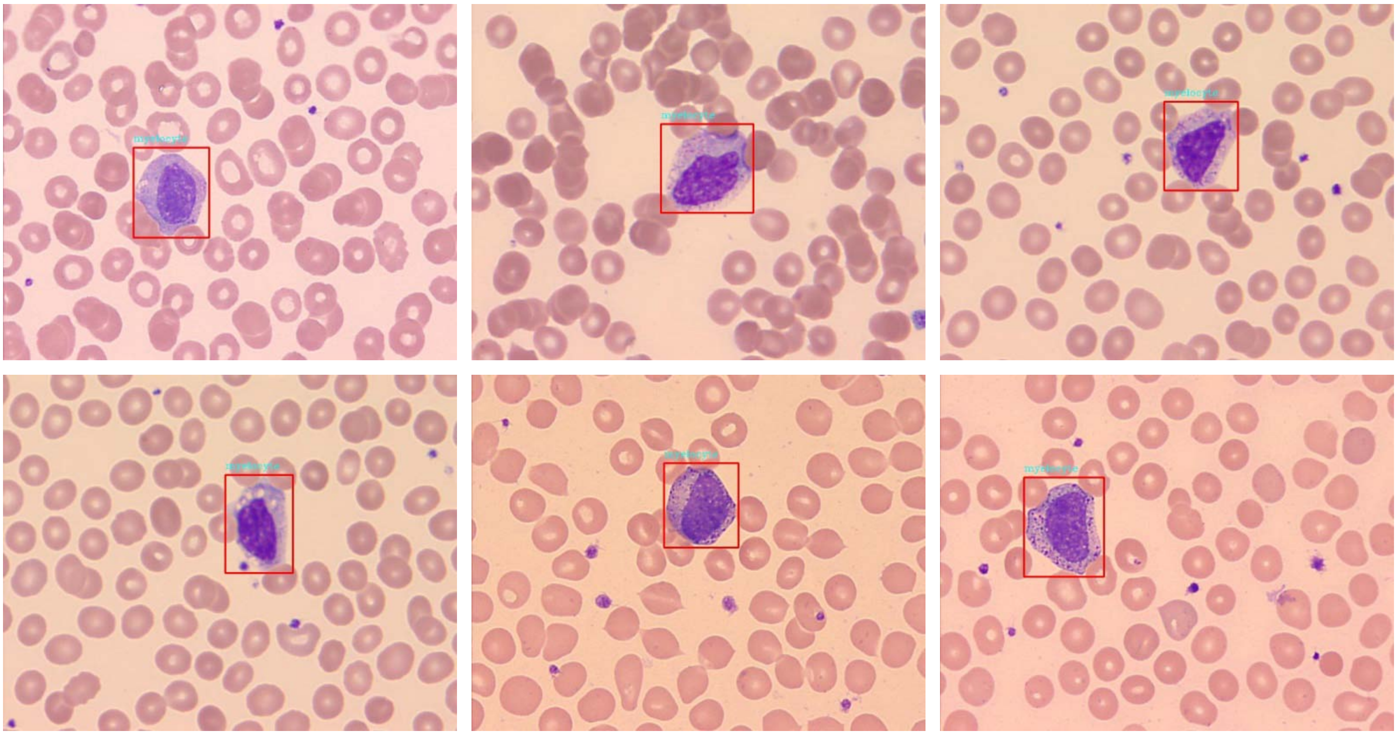

(C) detection results of myelocyte using SSD300×300_Smin=0.2 model

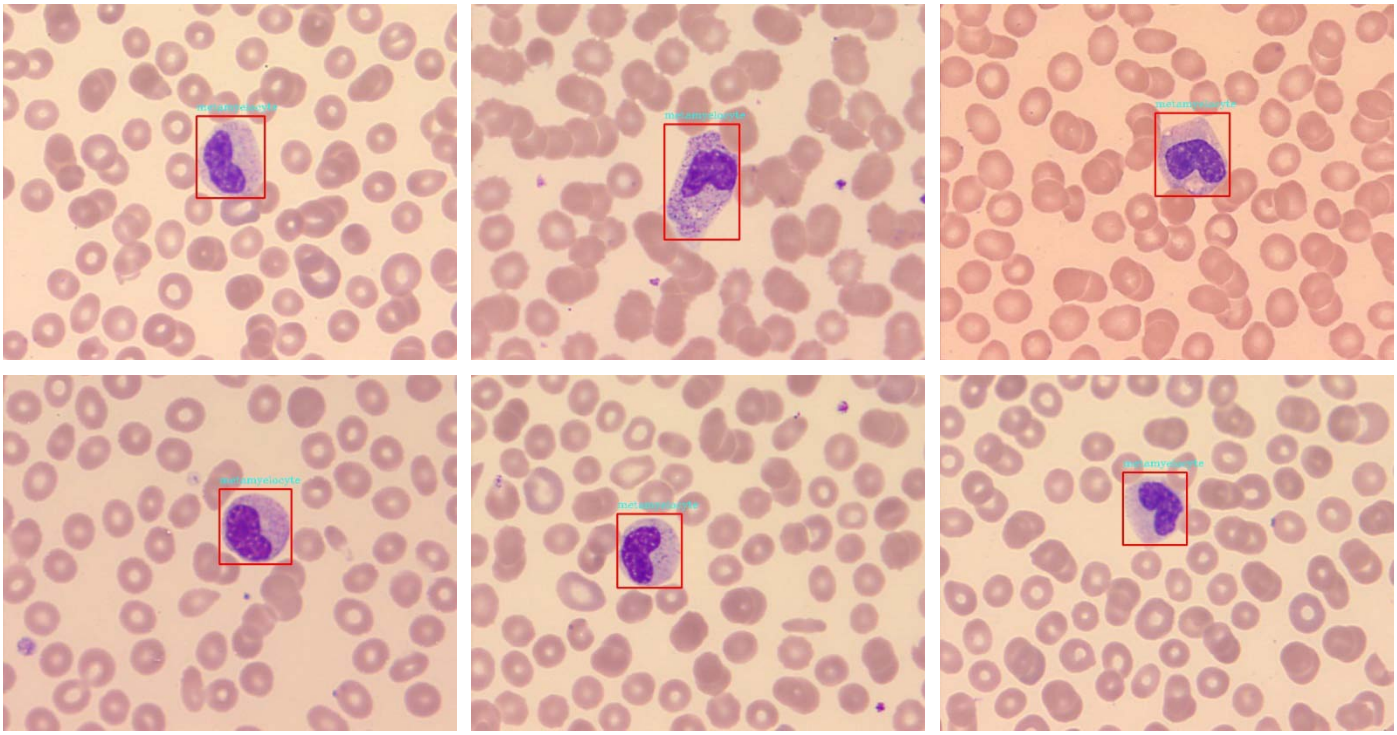

(D) detection results of metamyelocyte using SSD300×300_Smin=0.2 model

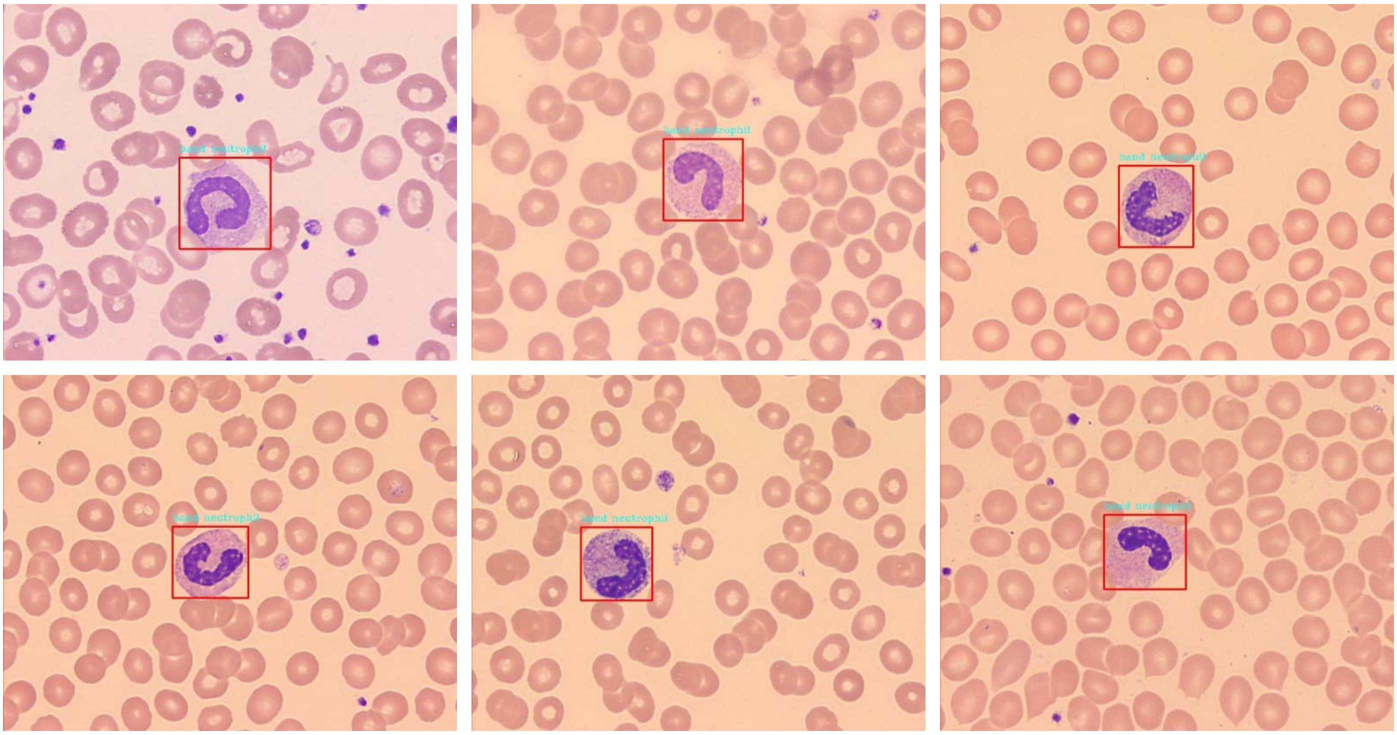

(E) detection results of band neutrophil using SSD300×300_Smin=0.2 model

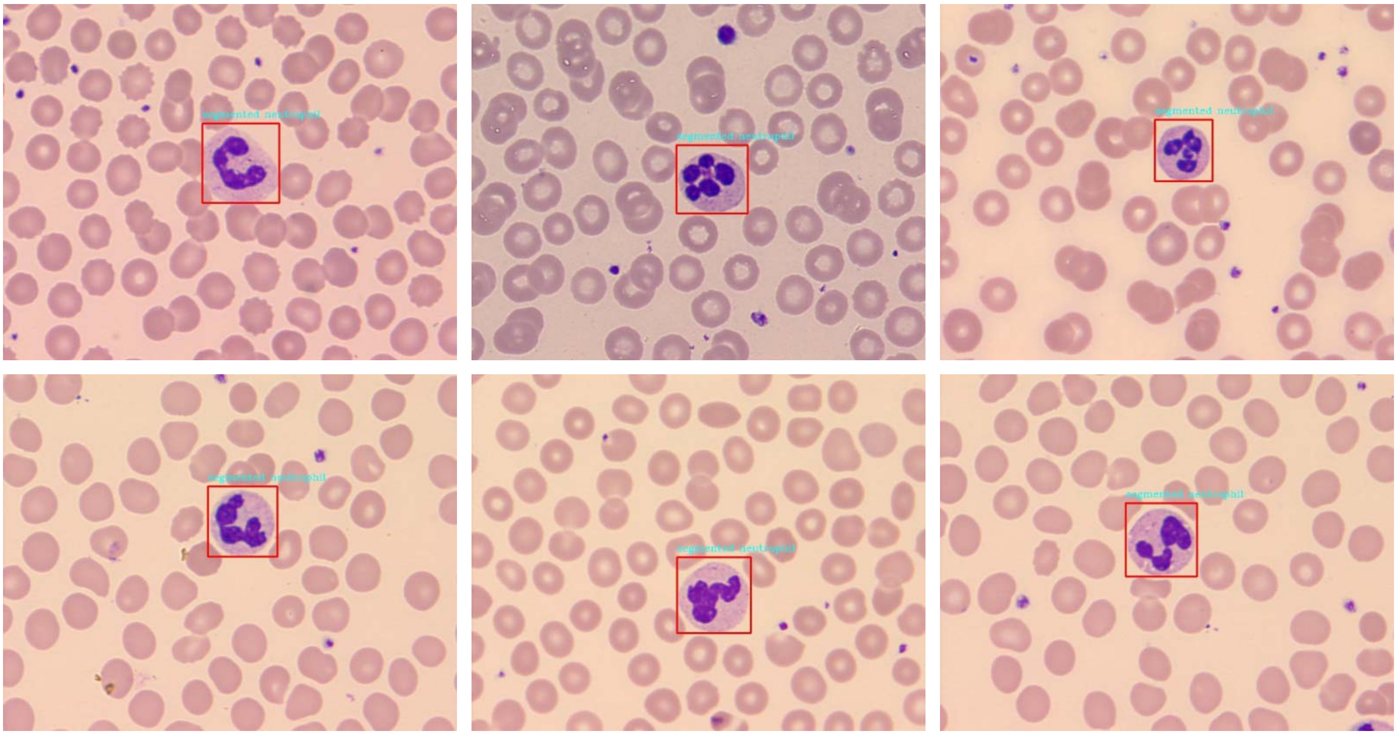

(F) detection results of segmented neutrophil using SSD300×300_Smin=0.2 model

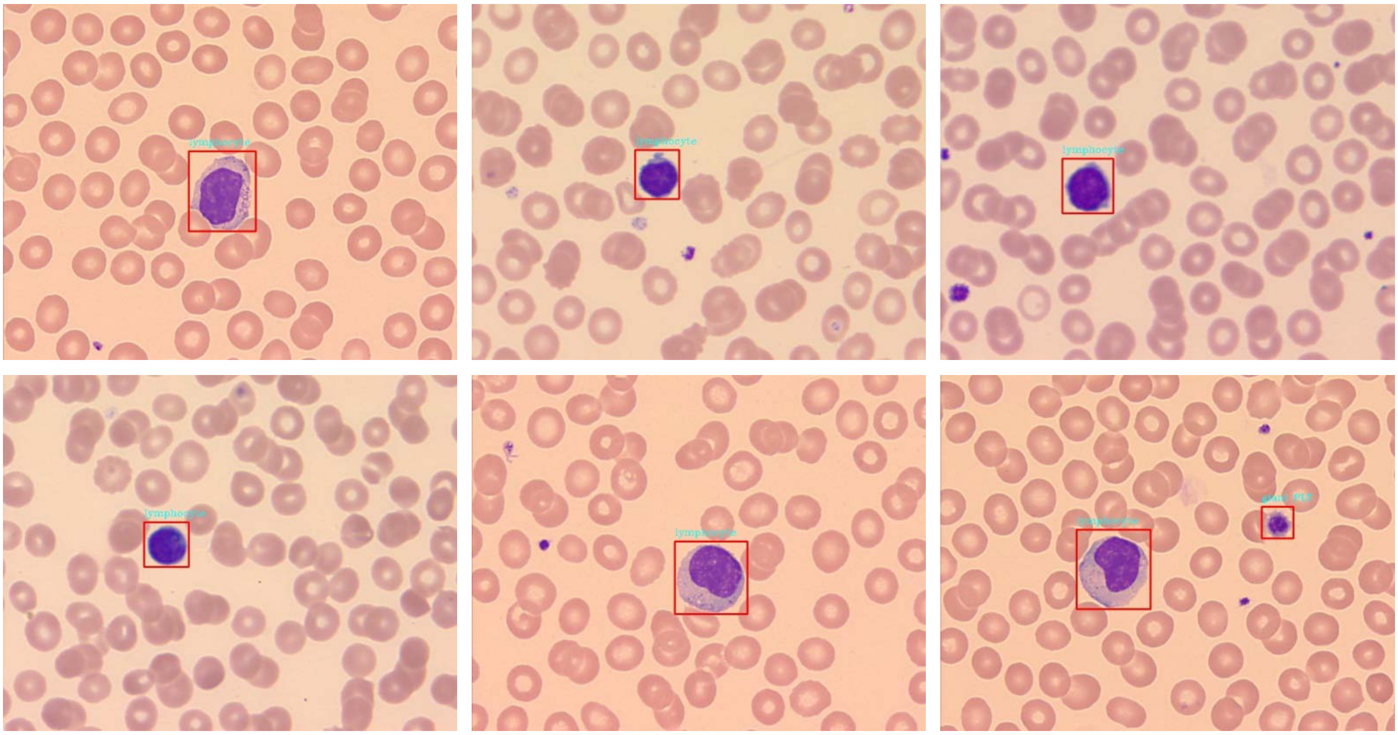

(G) detection results of lymphocyte using SSD300×300_Smin=0.2 model

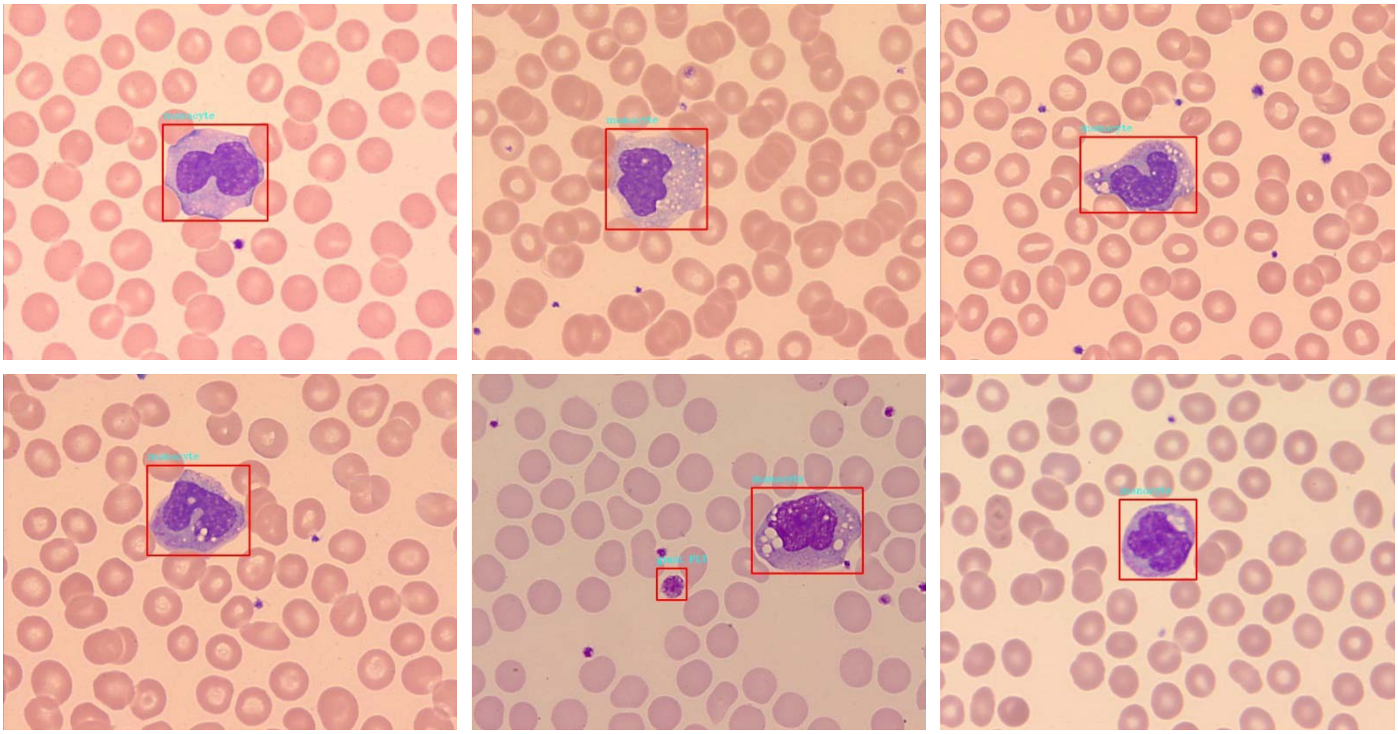

(H) detection results of monocyte using SSD300×300_Smin=0.2 model

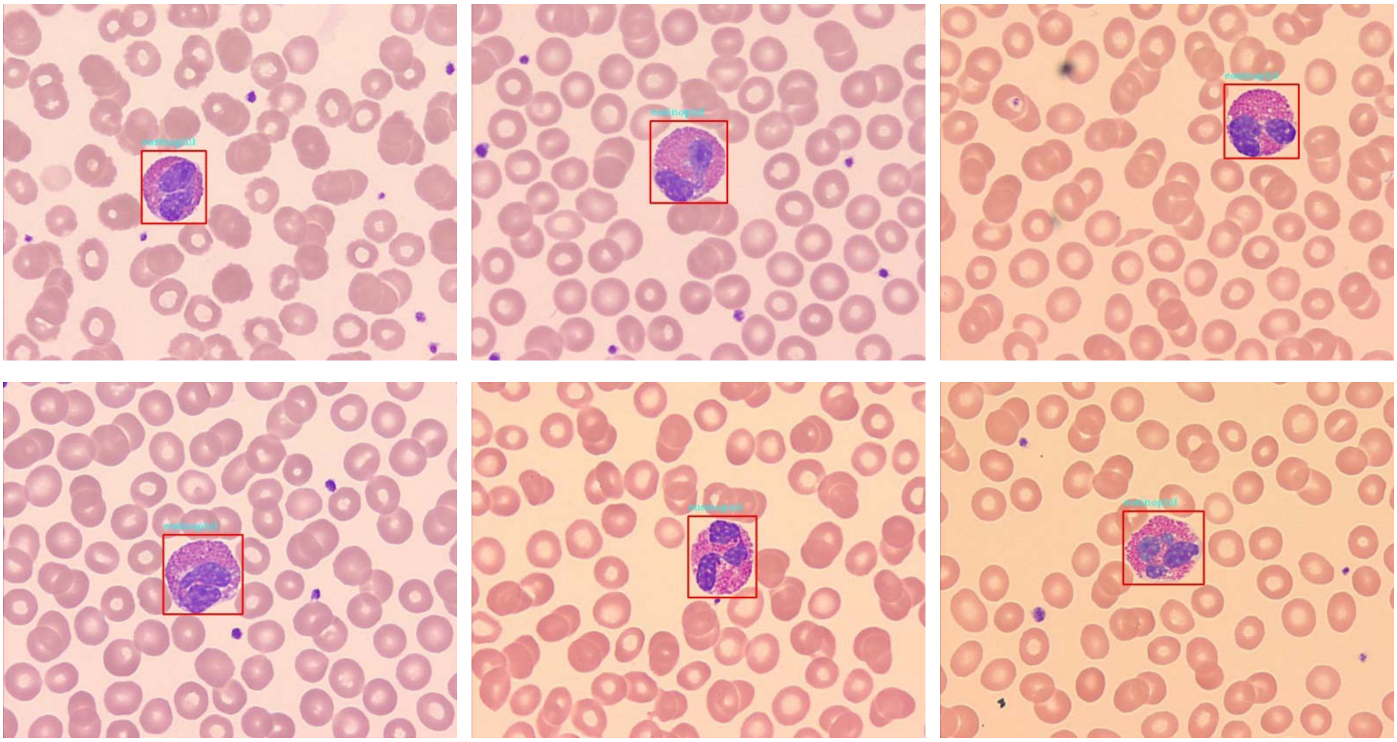

(I) detection results of eosinophil using SSD300×300_Smin=0.2 model

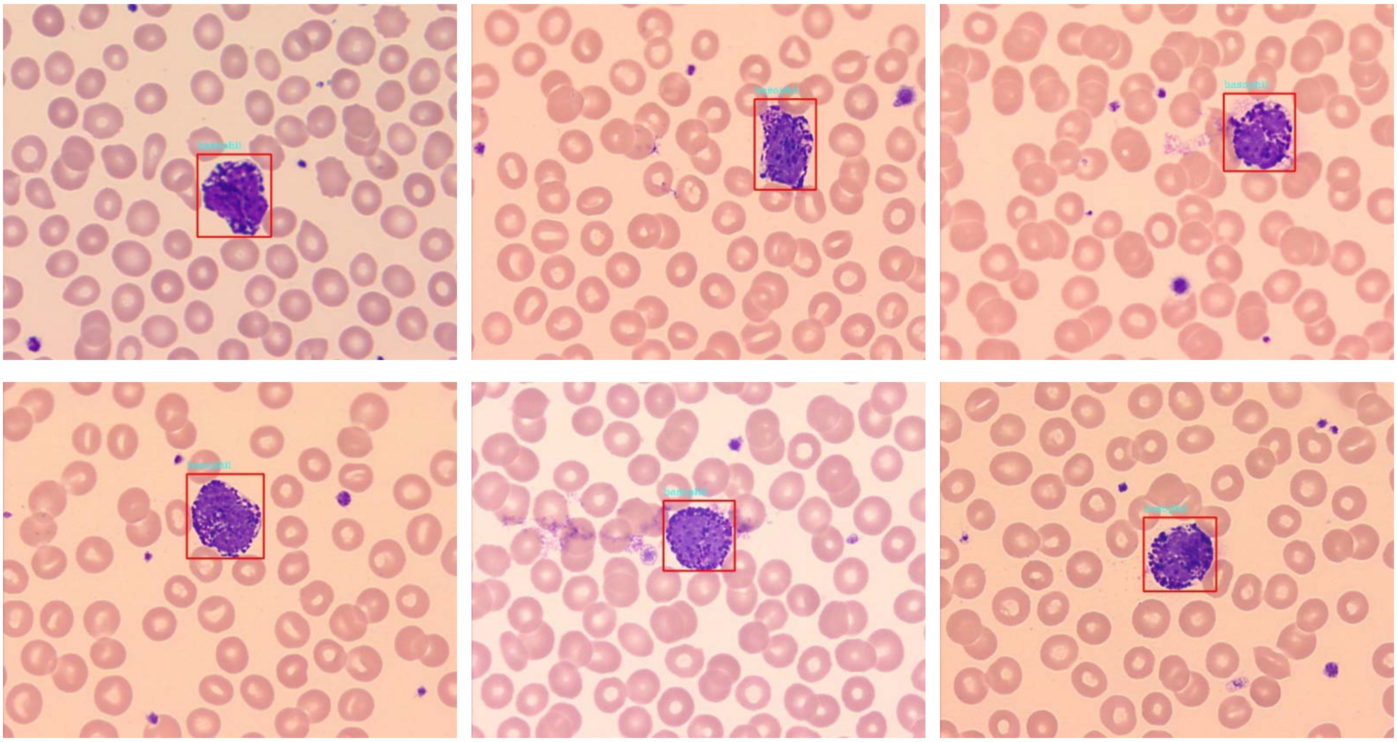

(J) detection results of basophil using SSD300×300_Smin=0.2 model

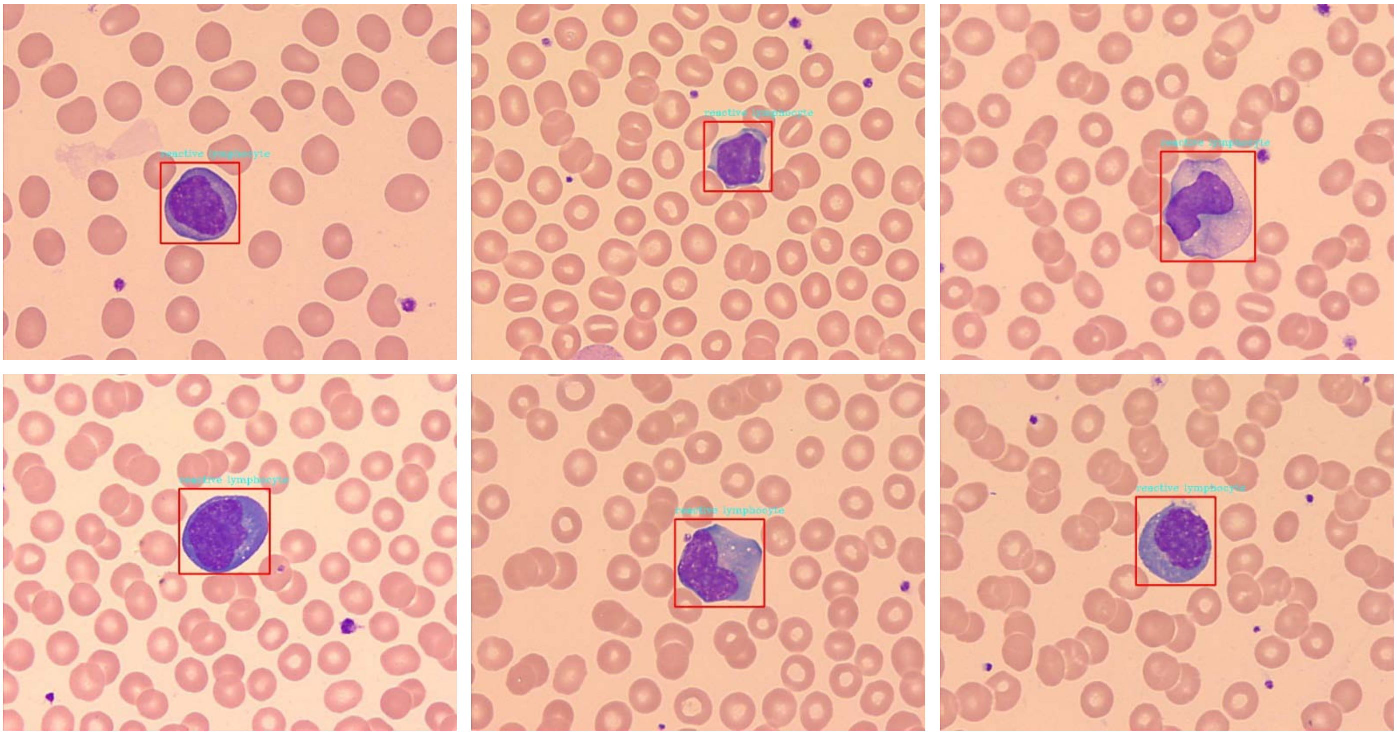

(K) detection results of reactive lymphocyte using SSD300×300_Smin=0.2 model

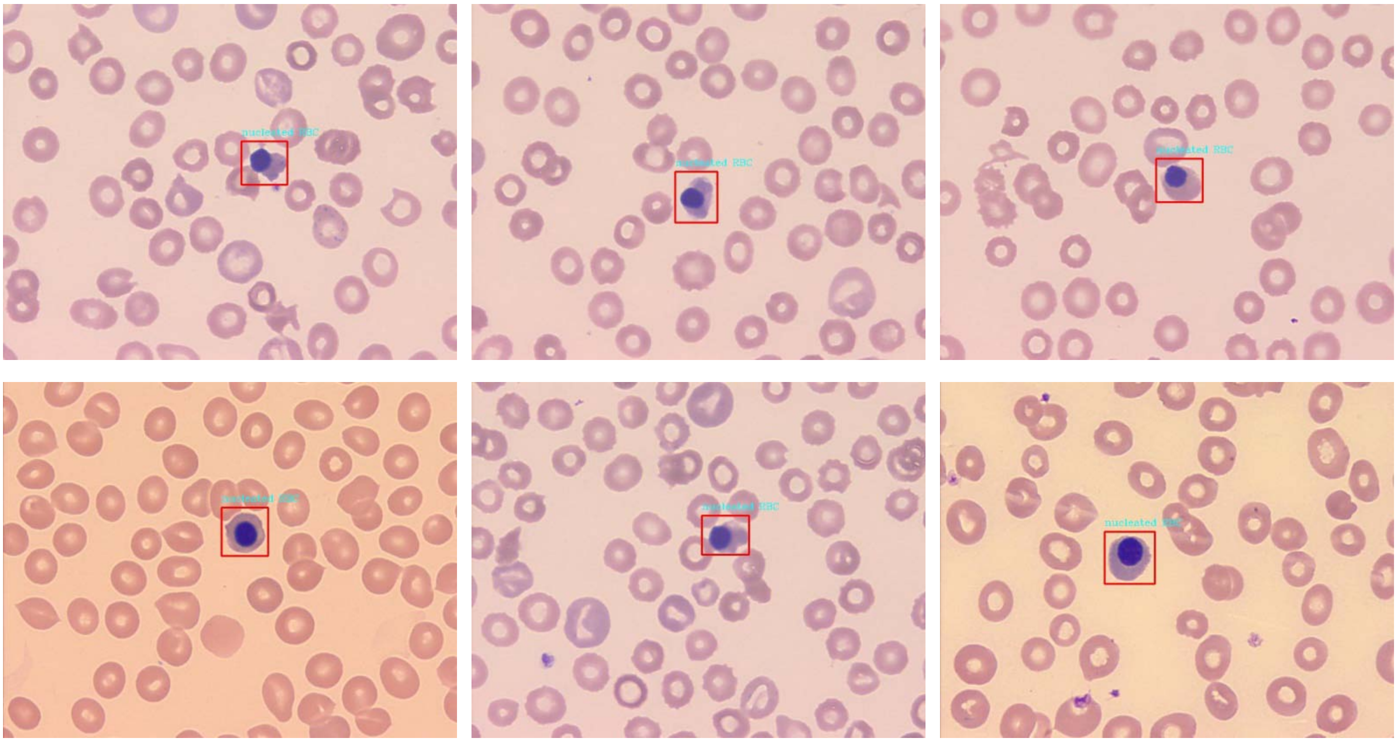

(L) detection results of small cells (NRBC) using SSD300×300_Smin=0.2 model)

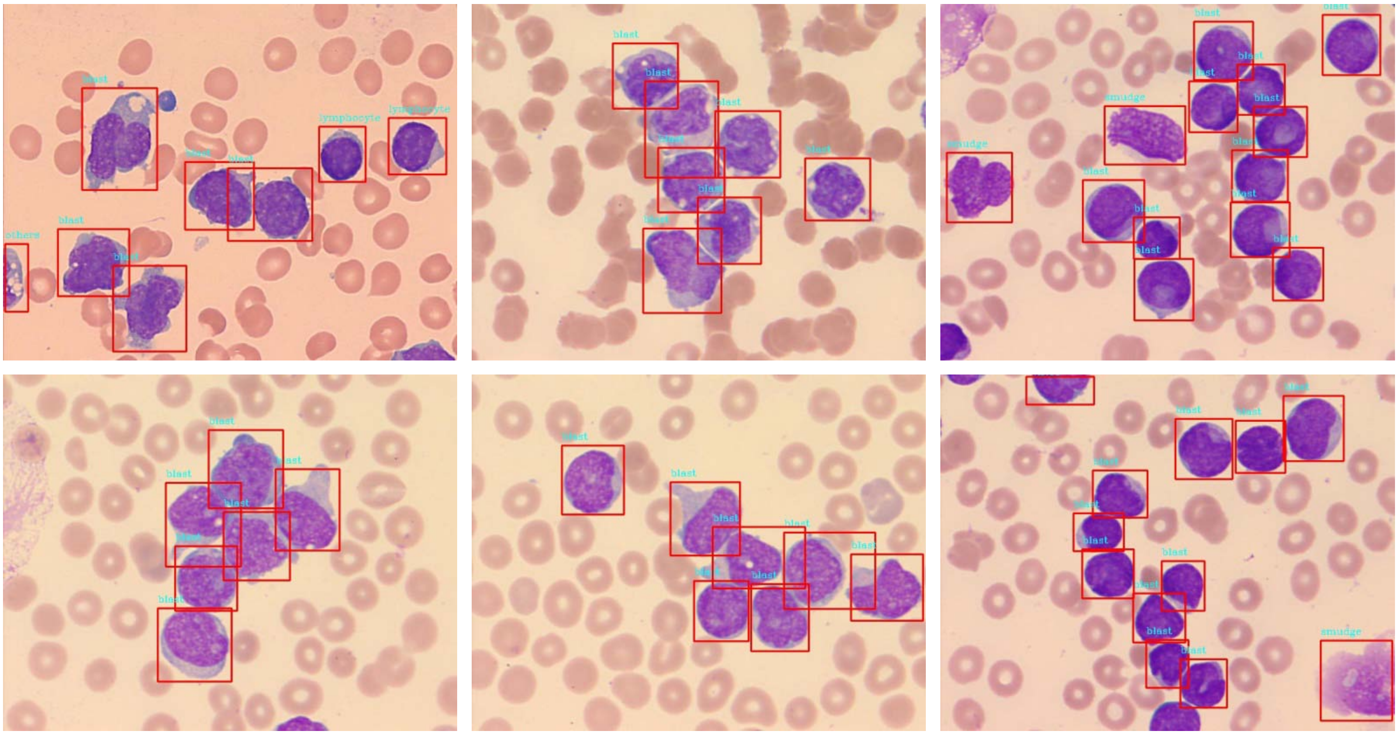

(M) detection results of dense scenes (linked cells) using SSD300×300_Smin=0.2 model

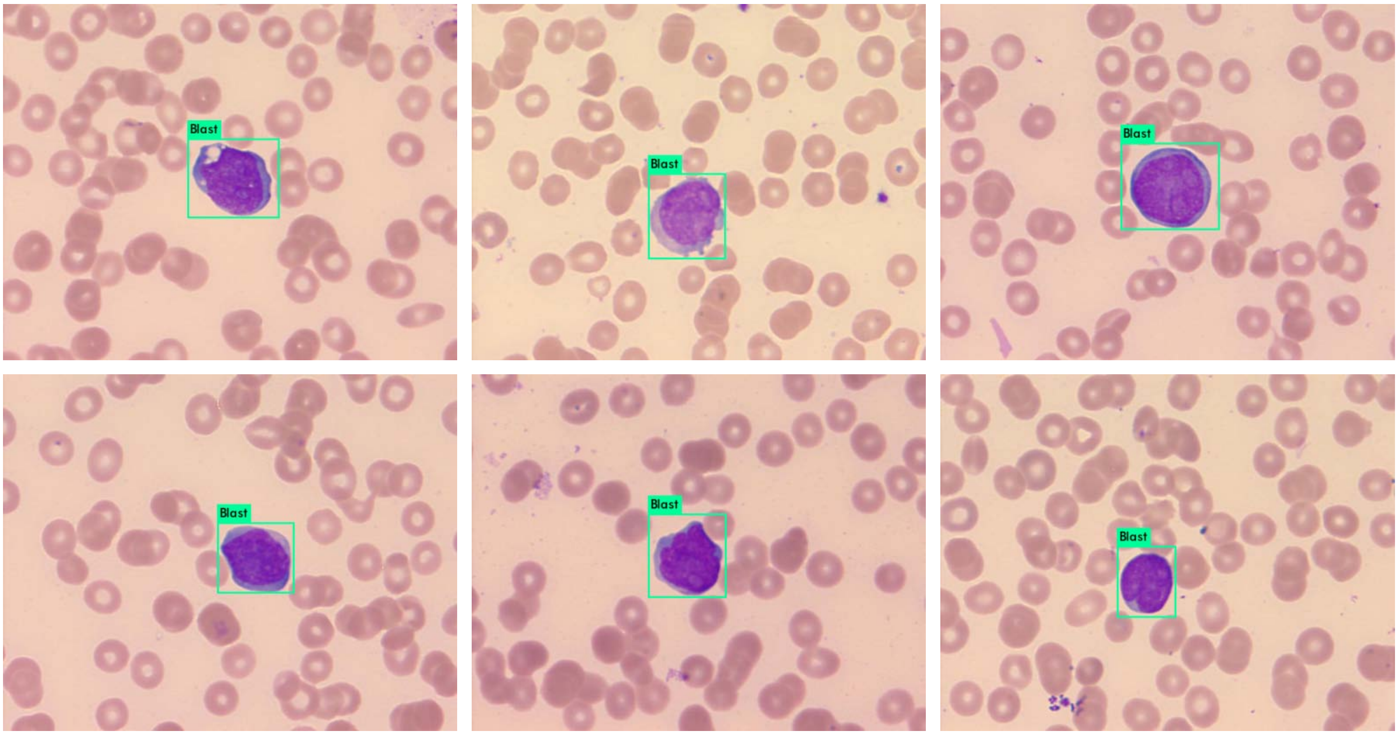

(N) detection results of blast using YOLOv3_320×320 model

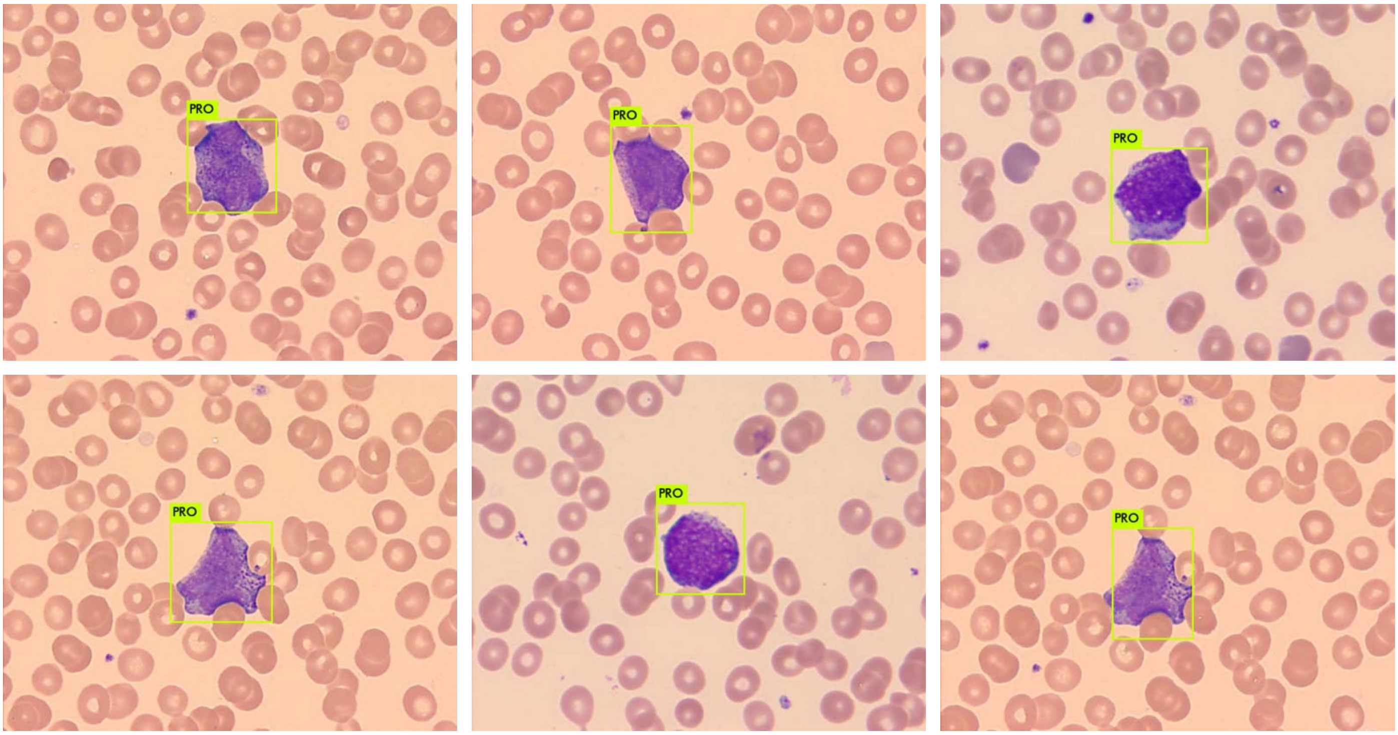

(O) detection results of promyelocyte using YOLOv3_320×320 model

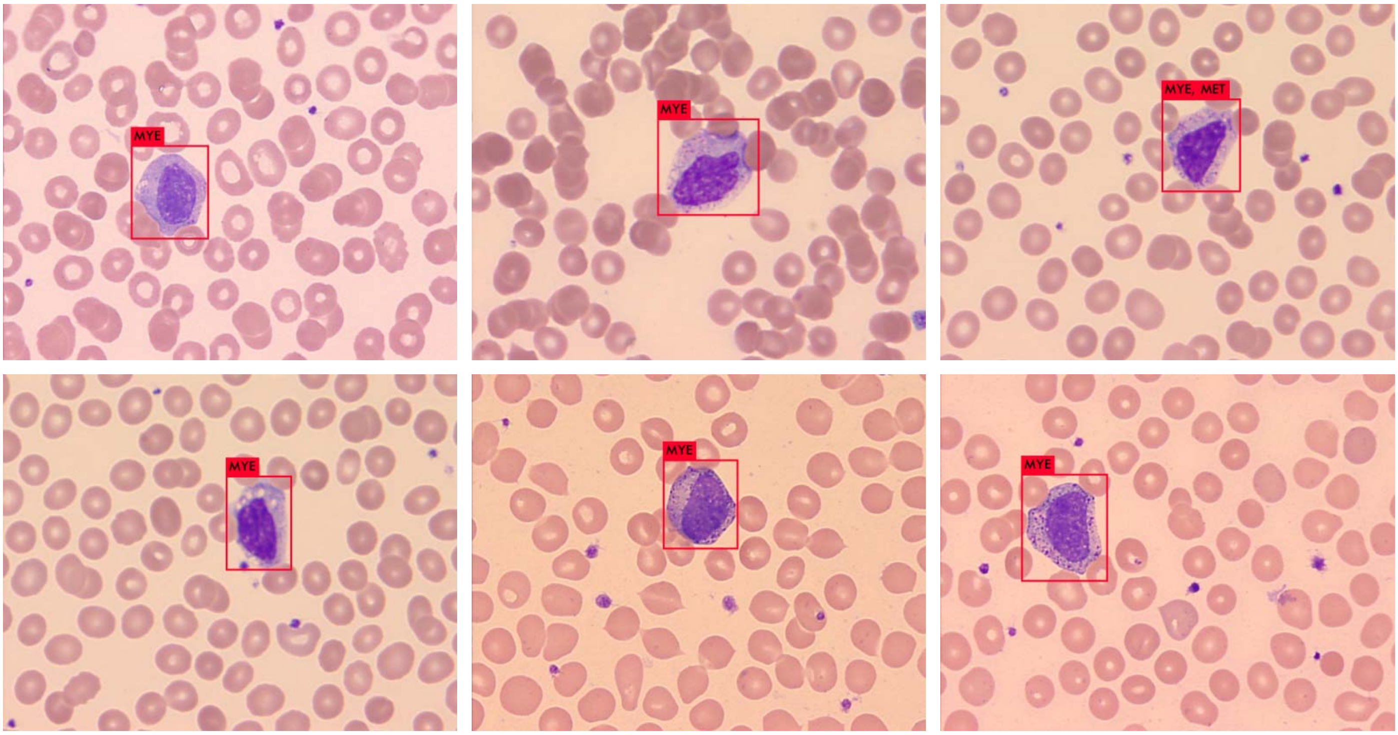

(P) detection results of myelocyte using YOLOv3_320×320 model

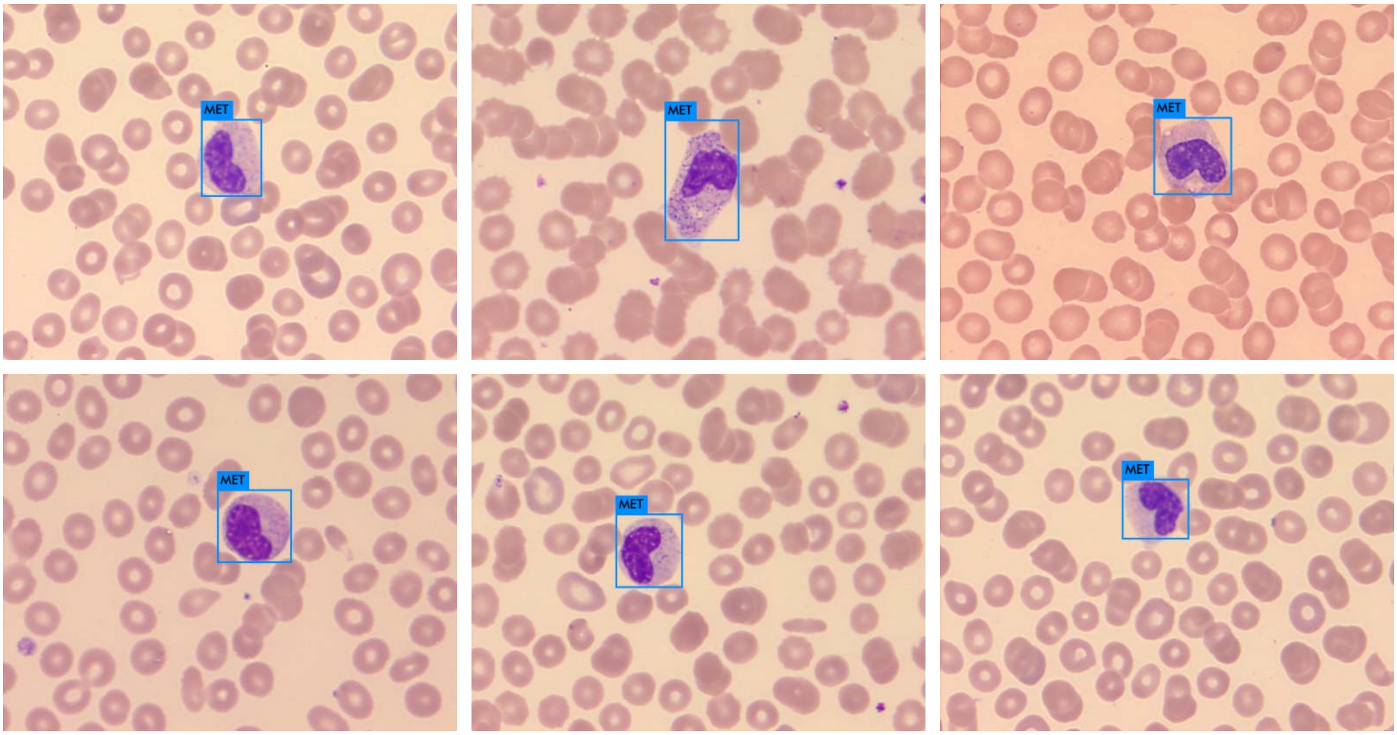

(Q) detection results of metamyelocyte using YOLOv3_320×320 model

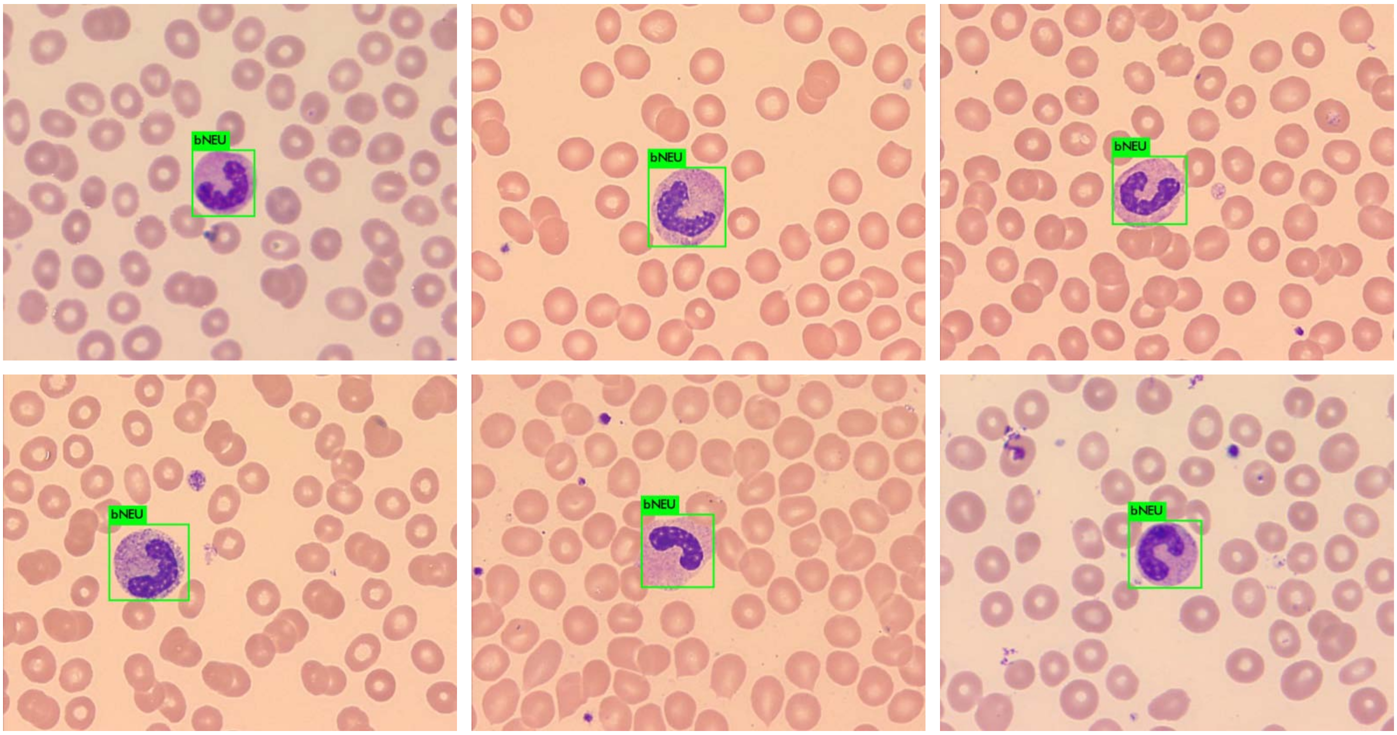

(R) detection results of band neutrophil using YOLOv3_320×320 model

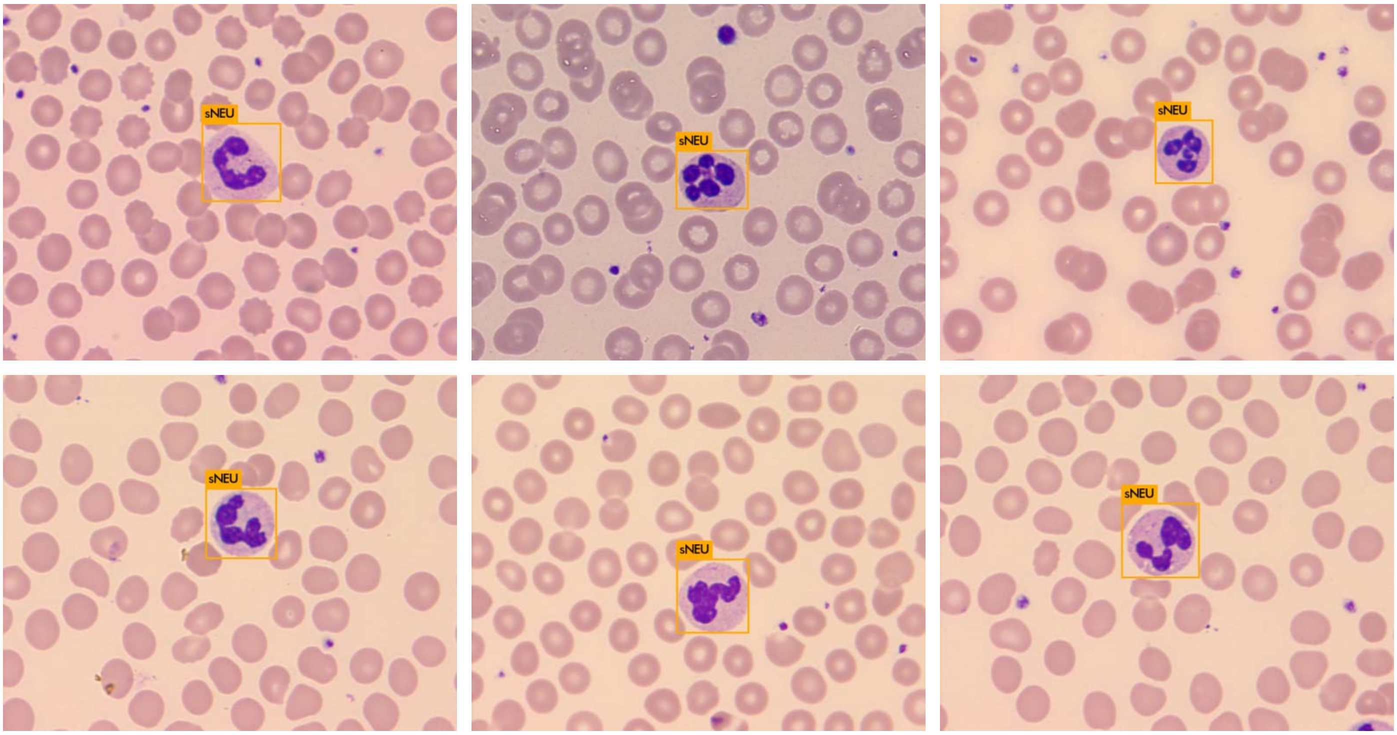

(S) detection results of segmented neutrophil using YOLOv3_320×320 model

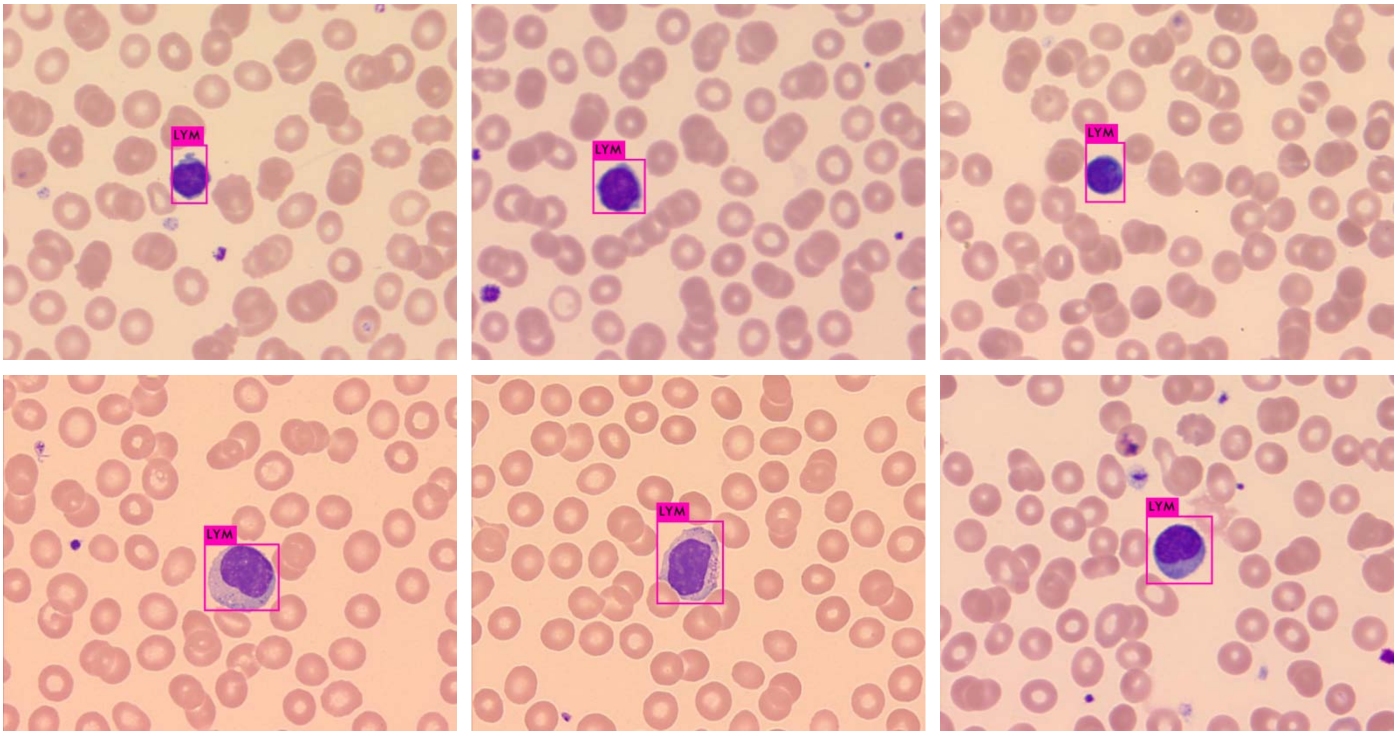

(T) detection results of lymphocyte using YOLOv3_320×320 model

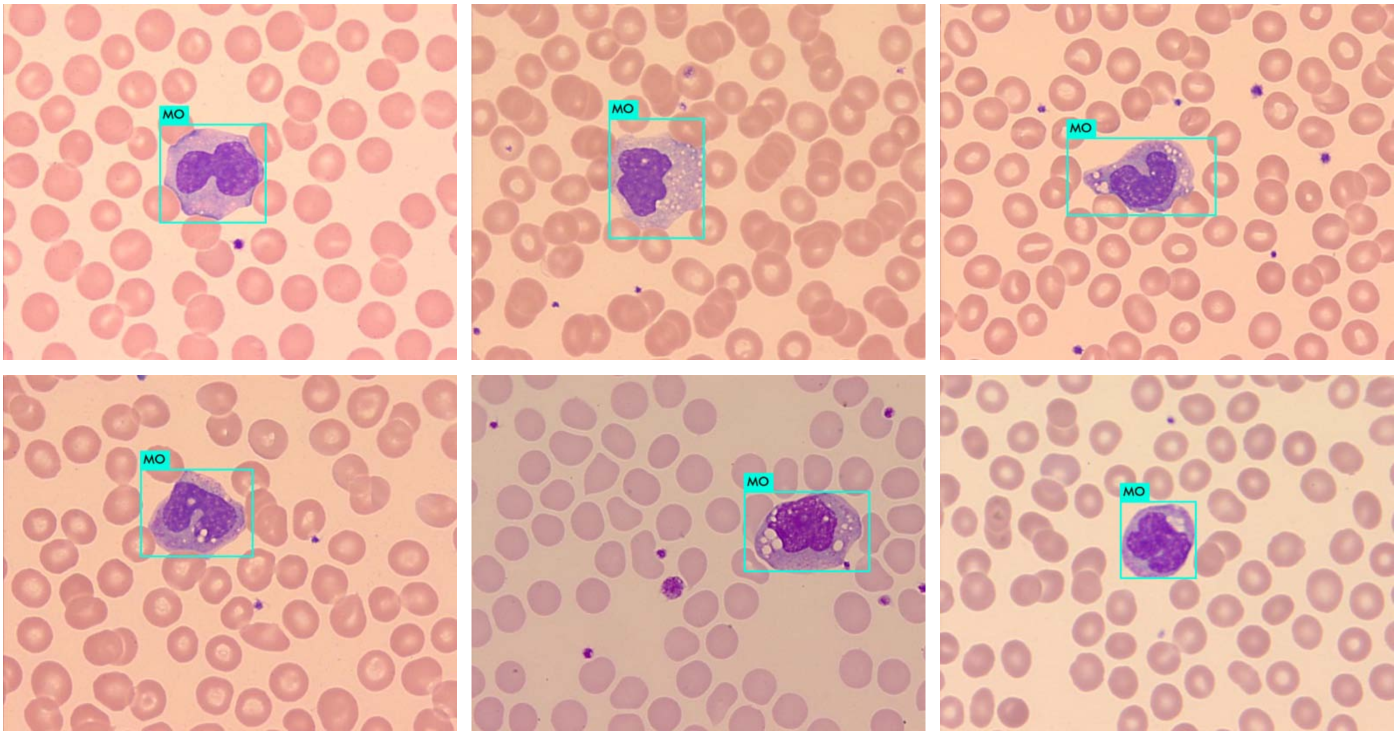

(U) detection results of monocyte using YOLOv3_320×320 model

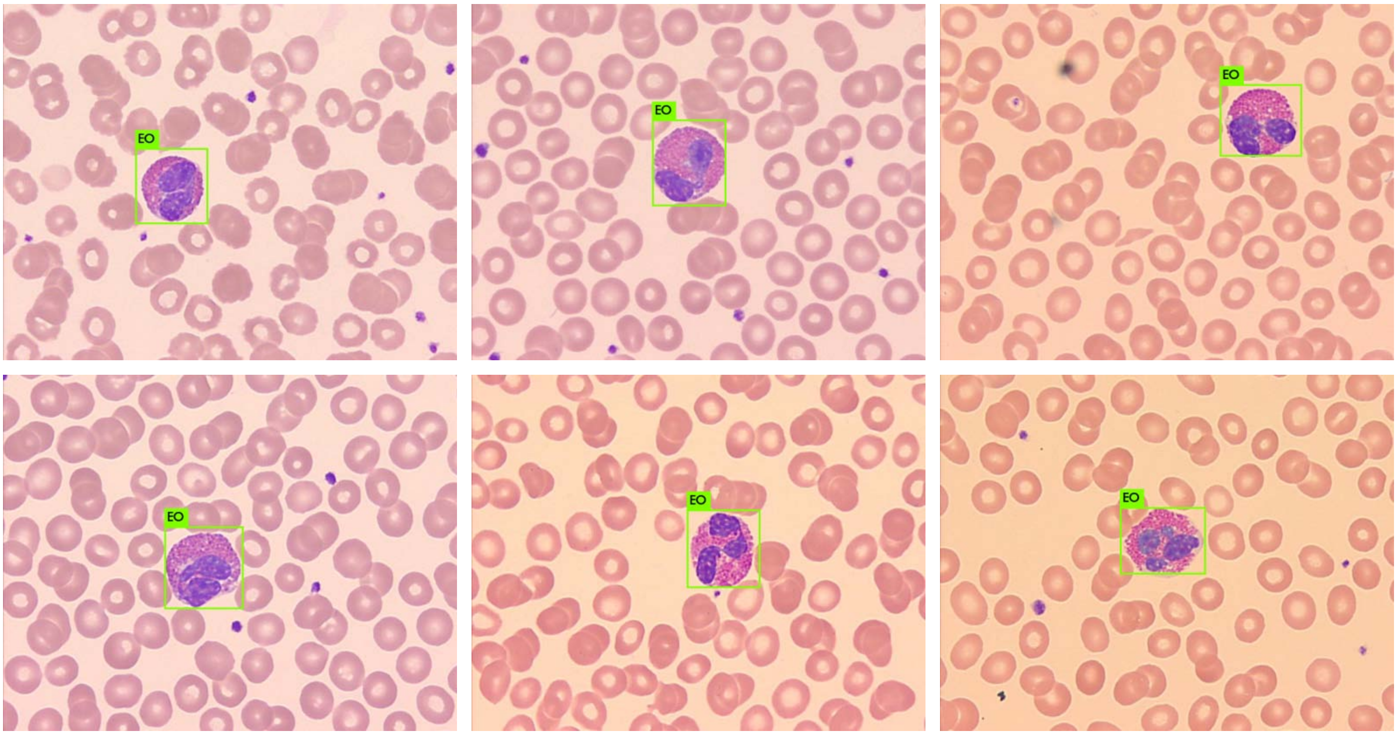

(V) detection results of eosinophil using YOLOv3_320×320 model

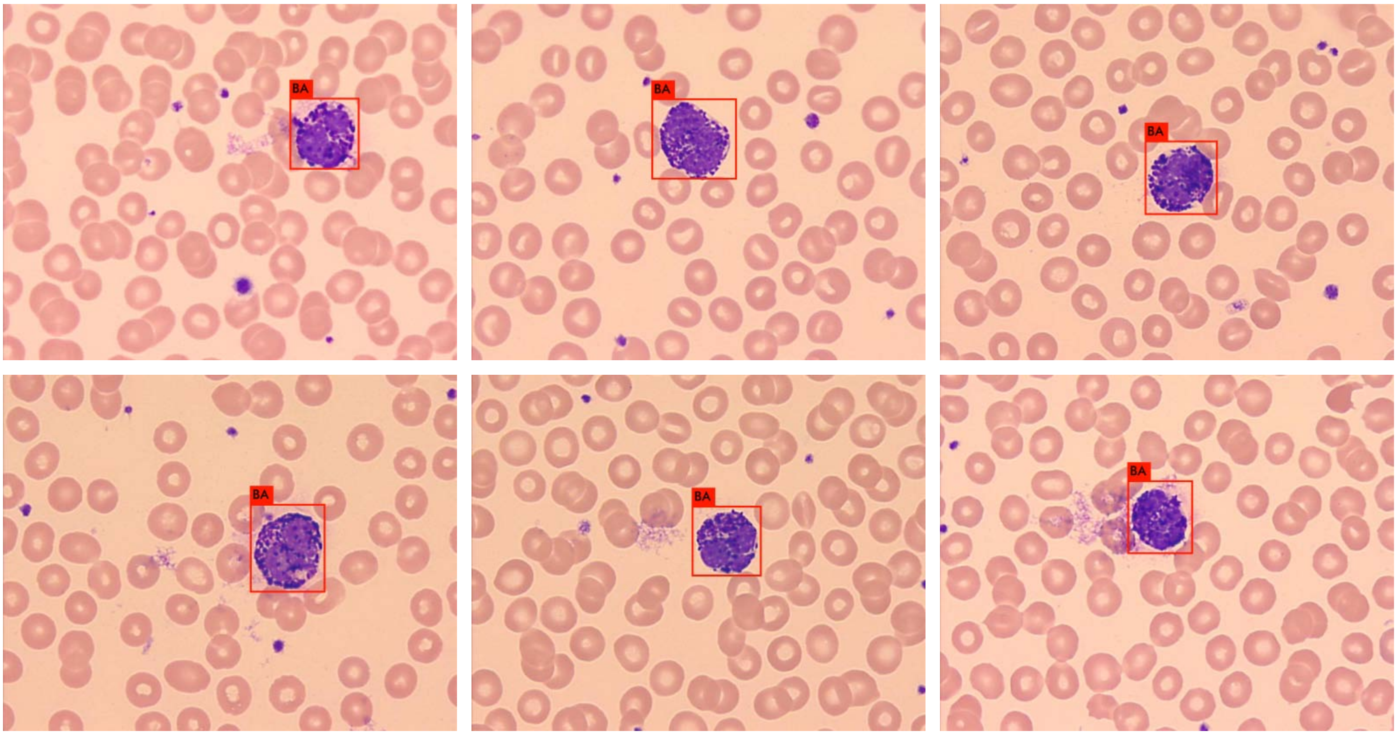

(W) detection results of basophil using YOLOv3_320×320 model

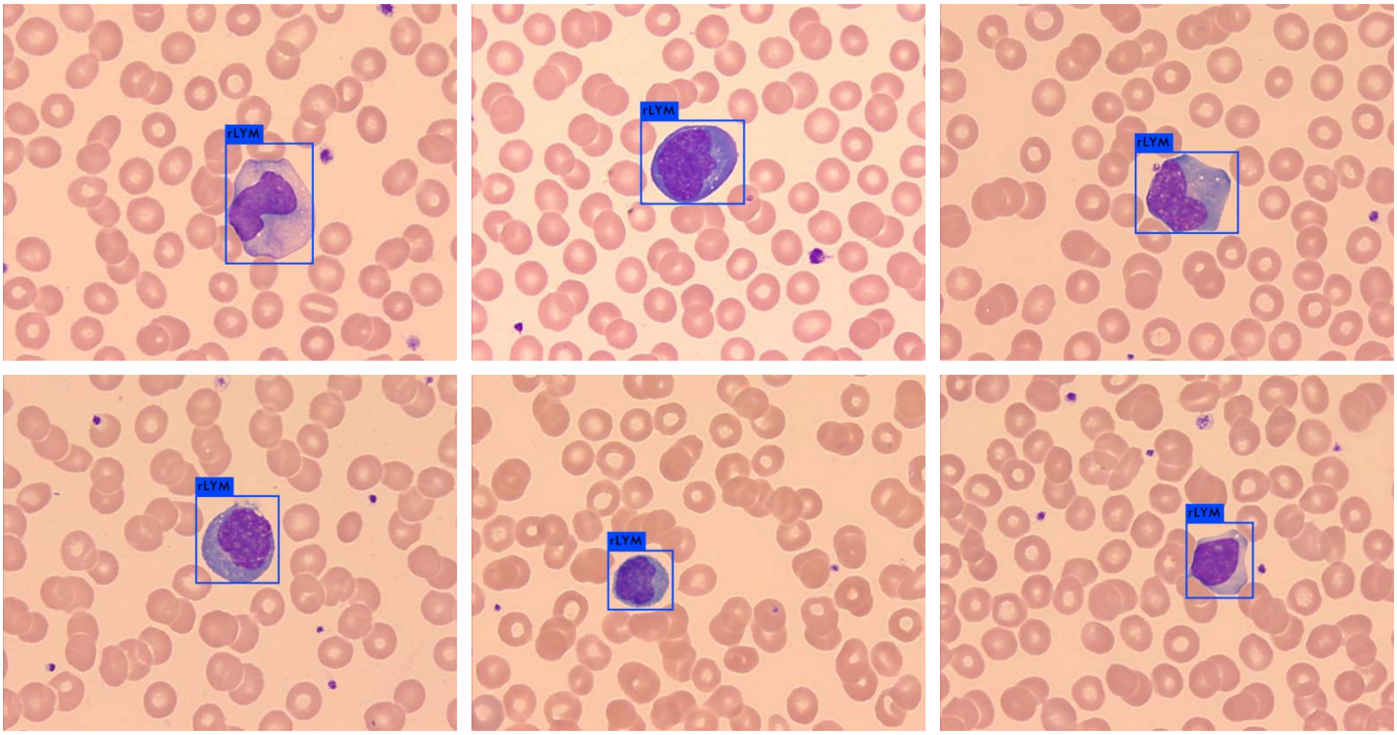

(X) detection results of reactive lymphocyte using YOLOv3_320×320 model

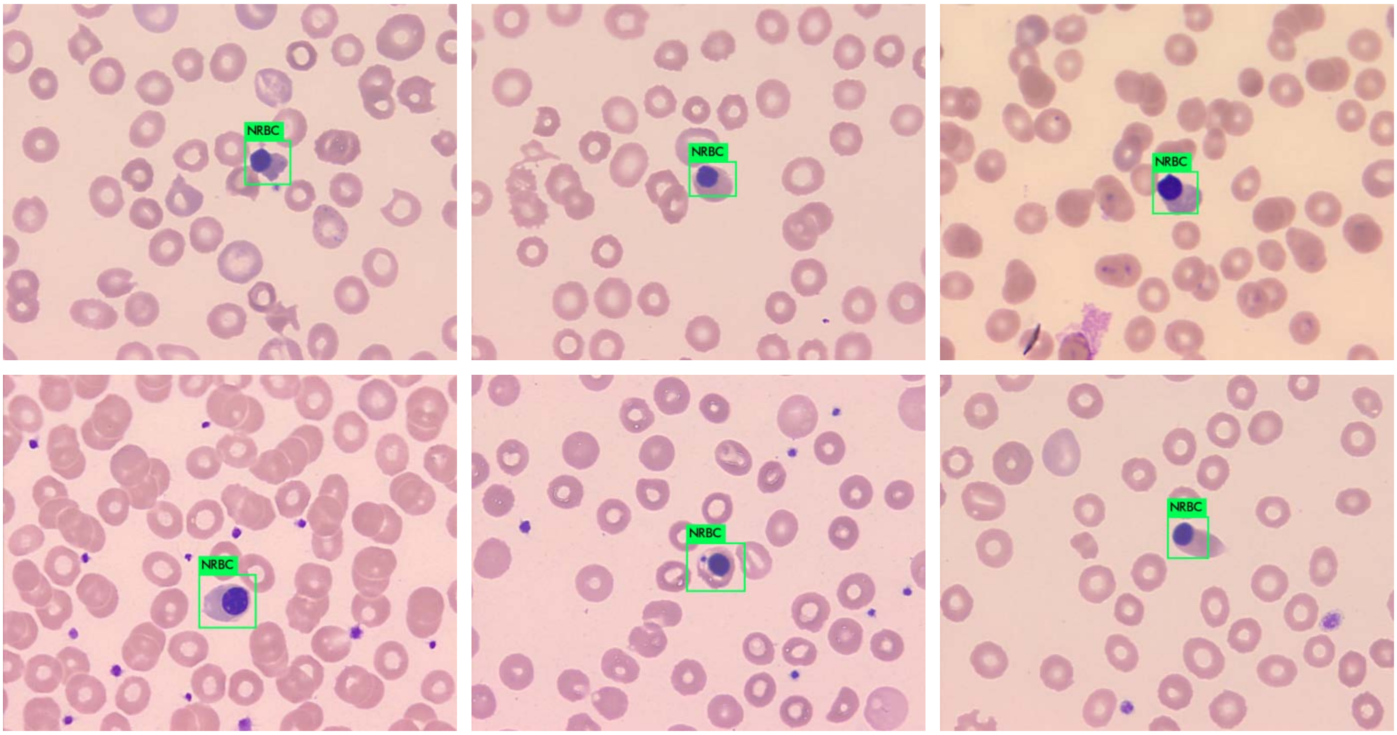

(Y) detection results of small cells (NRBC) using YOLOv3_320×320 model

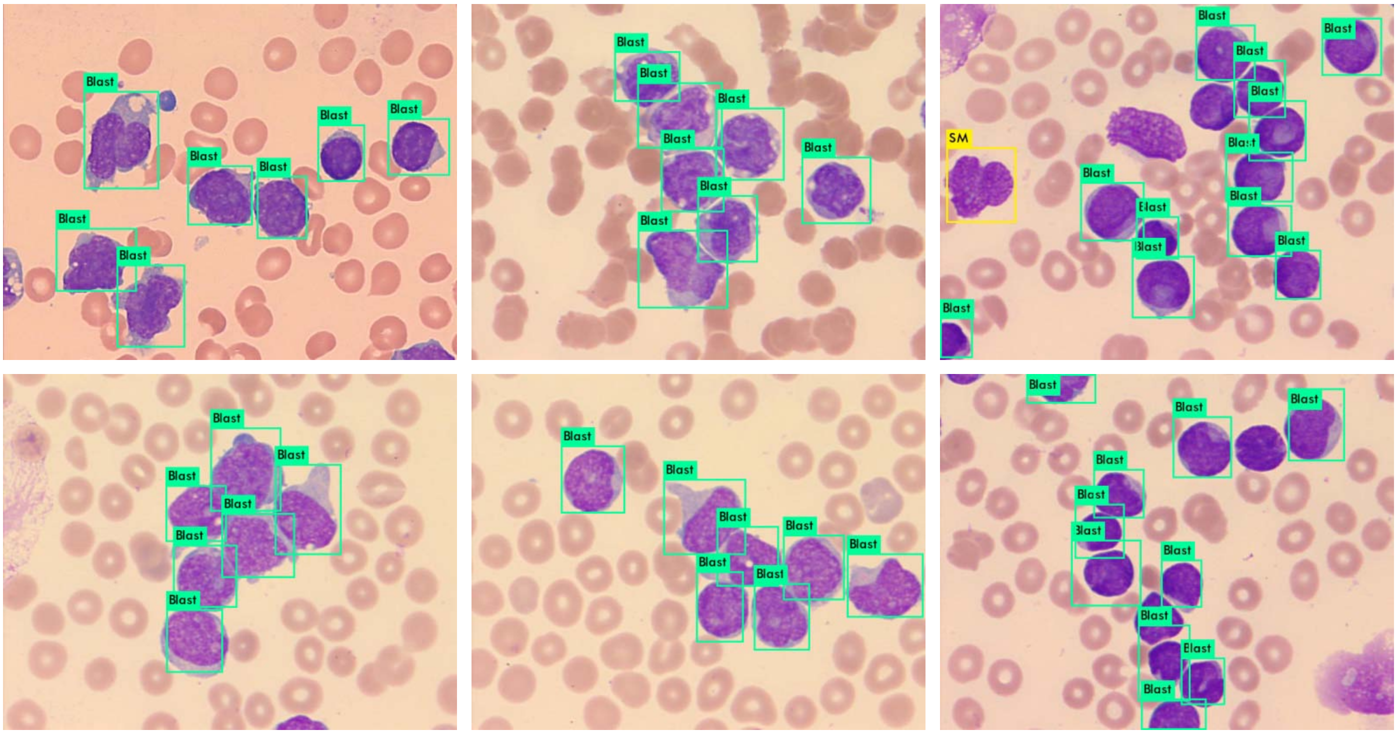

(Z) detection results of dense scenes (linked cells) using YOLOv3_320×320 model
